# Mapping the developing human cardiac endothelium at single-cell resolution identifies MECOM as a regulator of arteriovenous gene expression

**DOI:** 10.1093/cvr/cvac023

**Published:** 2022-02-25

**Authors:** Ian R McCracken, Ross Dobie, Matthew Bennett, Rainha Passi, Abdelaziz Beqqali, Neil C Henderson, Joanne C Mountford, Paul R Riley, Chris P Ponting, Nicola Smart, Mairi Brittan, Andrew H Baker

**Affiliations:** Centre for Cardiovascular Science, University of Edinburgh, Edinburgh EH16 4TJ, UK; Department of Physiology, Anatomy, and Genetics, University of Oxford, Oxford OX1 3PT, UK; Centre for Inflammation Research, University of Edinburgh, Edinburgh EH16 4TJ, UK; Centre for Cardiovascular Science, University of Edinburgh, Edinburgh EH16 4TJ, UK; Centre for Cardiovascular Science, University of Edinburgh, Edinburgh EH16 4TJ, UK; Centre for Cardiovascular Science, University of Edinburgh, Edinburgh EH16 4TJ, UK; Centre for Inflammation Research, University of Edinburgh, Edinburgh EH16 4TJ, UK; MRC Human Genetics Unit, Institute of Genetics and Cancer, University of Edinburgh, Edinburgh EH4 2XU, UK; Scottish National Blood Transfusion Service, Edinburgh EH14 4BE, UK; Department of Physiology, Anatomy, and Genetics, University of Oxford, Oxford OX1 3PT, UK; MRC Human Genetics Unit, Institute of Genetics and Cancer, University of Edinburgh, Edinburgh EH4 2XU, UK; Department of Physiology, Anatomy, and Genetics, University of Oxford, Oxford OX1 3PT, UK; Centre for Cardiovascular Science, University of Edinburgh, Edinburgh EH16 4TJ, UK; Centre for Cardiovascular Science, University of Edinburgh, Edinburgh EH16 4TJ, UK; Cardiovascular Research Institute Maastricht (CARIM), Maastricht University Medical Center, 6229 HX Maastricht, The Netherlands

**Keywords:** Human cardiac development, Single-cell RNA sequencing, Endothelial heterogeneity, Coronary vasculature formation, MECOM, Vascular regeneration

## Abstract

**Aims:**

Coronary vasculature formation is a critical event during cardiac development, essential for heart function throughout perinatal and adult life. However, current understanding of coronary vascular development has largely been derived from transgenic mouse models. The aim of this study was to characterize the transcriptome of the human foetal cardiac endothelium using single-cell RNA sequencing (scRNA-seq) to provide critical new insights into the cellular heterogeneity and transcriptional dynamics that underpin endothelial specification within the vasculature of the developing heart.

**Methods and results:**

We acquired scRNA-seq data of over 10 000 foetal cardiac endothelial cells (ECs), revealing divergent EC subtypes including endocardial, capillary, venous, arterial, and lymphatic populations. Gene regulatory network analyses predicted roles for *SMAD1* and *MECOM* in determining the identity of capillary and arterial populations, respectively. Trajectory inference analysis suggested an endocardial contribution to the coronary vasculature and subsequent arterialization of capillary endothelium accompanied by increasing *MECOM* expression. Comparative analysis of equivalent data from murine cardiac development demonstrated that transcriptional signatures defining endothelial subpopulations are largely conserved between human and mouse. Comprehensive characterization of the transcriptional response to MECOM knockdown in human embryonic stem cell-derived EC (hESC-EC) demonstrated an increase in the expression of non-arterial markers, including those enriched in venous EC.

**Conclusions:**

scRNA-seq of the human foetal cardiac endothelium identified distinct EC populations. A predicted endocardial contribution to the developing coronary vasculature was identified, as well as subsequent arterial specification of capillary EC. Loss of *MECOM* in hESC-EC increased expression of non-arterial markers, suggesting a role in maintaining arterial EC identity.

**See the editorial comment for this article ‘A new resource for human coronary vessel development’, by Ragini Phansalkar and Kristy Red-Horse, https://doi.org/10.1093/cvr/cvac094.**

## 1. Introduction

While the formation and homeostasis of the coronary vasculature is essential for heart muscle function, the molecular mechanisms underlying coronary vascular development remain incompletely understood. Previous studies using lineage-tracing tools in mouse have provided much needed insight into these mechanisms, including identifying the endocardium and sinus venosus (SV) as the two major sources of coronary vascular endothelium during cardiac development.^1,2^ A third source, the proepicardium, was previously proposed to contribute a minor population of coronary endothelial cells (ECs),^3,4^ although this notion has recently been challenged.^5^ Following the formation of the primitive coronary vascular plexus from these sources and onset of blood flow, subsequent remodelling occurs, giving rise to the distinct EC populations present in the mature vascular bed of the fully developed heart.^6,7^ Recent studies have elegantly mapped the remodelling of the immature coronary EC plexus in mouse cardiac development, including identification of a role for the transcription factor (TF) Dach1 in potentiating developmental arterial remodelling.^7–9^ However, given that these advances in our understanding of coronary vascular development primarily originate from murine lineage-tracing studies, the relevance of these findings for human cardiac development remains largely unknown.

Advances in single-cell RNA sequencing (scRNA-seq) have been instrumental in enhancing our understanding of embryonic development, permitting the objective mapping of underlying transcriptional changes at single-cell resolution. In addition, improvements in high-throughput scRNA-seq platforms have facilitated the characterization of tens of thousands of cells in parallel, thus allowing for ‘atlas’ studies to map the gene expression profile of entire organs during embryogenesis.^10^ In recent years, such scRNA-seq studies have mapped the transcriptional profile of both murine and human heart development.^11–19^ In the study by Cui *et al.*,^13^ scRNA-seq was conducted using cells isolated from specific regions of 18 human foetal hearts, ranging from 5 to 24 weeks gestation. Subsequent dimensionality reduction and clustering analysis revealed an EC cluster of 595 cells characterized by expression of endothelial markers, such as *PECAM1.*^13^ Similarly, a clear EC population was identified in a study from Suryawanshi *et al.*^12^ in which cells isolated from three healthy human foetal hearts (19–22 weeks) were processed using scRNA-seq. Both studies mapped the expression of known EC marker subtypes to allow annotation of clusters corresponding to endocardium, coronary vascular EC, and valvular EC. Nevertheless, the relatively low numbers of EC in these datasets prevented further characterization of cardiac EC subtypes, including the identification of distinct arterial, venous, capillary, and lymphatic populations. In addition, these low EC numbers also prevented the application of methods to infer the dynamic cellular changes accompanying cardiac EC development.

While scRNA-seq studies of the developing mouse heart yielded large numbers of EC in their datasets,^11,16–18^ their analysis focused on other cell types, such as the cardiac conduction system, with minimal interpretation of EC heterogeneity and potential function. These included a study from Goodyer *et al.*,^15^ which analysed distinct vascular EC and endocardial cell populations from E16.5 mouse hearts.

In this study, we used scRNA-seq to comprehensively map the transcriptional signature of over 10 000 human foetal cardiac ECs isolated by fluorescence activated cell sorting (FACS) from two human foetal hearts at 13- and 14-weeks’ gestation. Unsupervised clustering, gene regulatory analysis, and trajectory inference methods revealed the transcriptional profile of heterogeneous EC populations and predicted dynamic cellular changes including arterial EC specification. In addition, we functionally validated MECOM as a regulator of arterial EC identity, thereby demonstrating the suitability of our novel scRNA-seq dataset to make *in-silico* predictions, capable of informing future strategies to guide endothelial identity. Collectively, findings from this study complement and expand upon knowledge previously obtained from murine development, bringing insights into human EC heterogeneity and pathways determining specification of subpopulations that are essential for understanding human coronary vascular formation.

## 2. Methods

### 2.1 Tissue collection and study approval

Human foetal cardiac tissue was acquired following elective termination of pregnancy. Informed written parental consent was obtained from all participants. Tissue was not collected in cases where termination of pregnancy was conducted due to an identified foetal or pregnancy abnormality. Ethical approval for the collection of foetal tissue was performed in accordance with all relevant guidelines and following study approval from the Lothian Research Ethics Committee (Study code: 08/1101/1) and the Research and Development Office (Study code: 2007/R/RM/10). This study was performed in accordance with the Declaration of Helsinki.

### 2.2 Isolation of foetal cardiac ECs

Cardiac ECs were isolated from the ventricular tissue of freshly collected human foetal hearts using a method adapted from van Beijnum *et al.*^20^ Digestion was performed at 37°C using a digestion solution containing 9 mL 0.1% collagenase II and 1 mL of 2.5 U/mL dispase. Then, 75 µL of 1 mg/mL DNaseI was added following 20 min incubation prior to a further 15 min incubation at 37°C. Digestion was quenched by the addition of 10 mL cold RPMI with 10% FCS and undigested clumps of tissue removed using a 100 µM cell strainer. Red blood cell lysis was performed by incubating cells for 2 min in red blood cell lysis buffer at room temperature prior to neutralizing with RPMI + 0.1% BSA. Cells were stained on ice for 45 min with APC anti-human CD31 and PE anti-human CD45 (Supplementary material online, *Table S1*). CD31+ CD45− ECs were isolated by FACS with DAPI staining being used to allow exclusion of dead cells.

### 2.3 scRNA-seq of foetal cardiac ECs

Sorted CD31+ CD45− ECs were counted manually using a haemocytometer with trypan blue staining used to identify non-viable cells. Viability exceeded 85% for both samples. A total of 8000 cells were loaded onto the 10X Chromium controller and library construction conducted using the Single Cell 3’ Reagent Kit (V3.1) in accordance with the manufacturer’s instructions. Libraries were sequenced using the Illumina NovaSeq 6000 platform.

### 2.4 scRNA-seq data analysis

Raw-sequencing data was processed using the 10X CellRanger pipeline (Version 3.1.0.) aligning reads to the GRCh38-3.0.0 genome reference. Barcodes corresponding to cells were distinguished from those corresponding to empty droplets using both the DropUtils package^21^ and the default cell calling method applied within the CellRanger pipeline. Cells with a total UMI count exceeding three median absolute deviations (MADs) from the median value were removed from downstream analysis using the R Scater package.^22^ Similarly, cells with a high proportion of counts from mitochondrial genes (>3 MADs) or with a low total gene count (<2 MADs) were also excluded. Data normalization was performed using the MultiBatchNormalisation method^23^ prior to merging datasets. Normalized count data were then scaled, and principal component analysis (PCA) applied using genes with the most variable expression across the combined dataset.^24^ Following batch correction using Harmony,^25^ non-supervised clustering was performed, and data visualized using Uniform Manifold Approximation and Projection (UMAP).^24^ A small cluster (155 cells) characterized by increased expression of fibroblast/smooth muscle cell markers (*ACTA2* and *MYH11*) and reduced EC marker expression (*PECAM1* and *CDH5*) was removed from the dataset prior to rerunning data normalization, PCA, and data visualization. Significantly differentially expressed genes (DEGs) within individual clusters were identified using the Wilcoxon signed-rank test (Bonferroni corrected *P*-value <0.05) and a minimum log_*e*_(fold change) threshold of 0.3.^24^ Additionally, only DEGs expressed in more than 30% of cells within their corresponding cluster were retained for further analysis.

Enriched metagene signatures were identified using the R package SCRAT v1.0.0.^26^ Gene regulatory analysis was performed using the standard R Single-Cell rEgulatory Network Inference and Clustering (SCENIC) workflow.^27^ RNA velocity analysis was conducted using the python package scVelo^28^ with the stochastic model being applied to predict the direction and magnitude of cellular dynamics. Trajectory inference tool Slingshot was performed using the standard workflow.^29^ Genes significantly differentially expressed over pseudotime were identified using the TradeSeq package^30^ with the top 2000 most variably expressed genes in the dataset being used to fit the negative binomial generalized additive model.

### 2.5 Human embryonic stem cell-derived EC differentiation and siRNA-mediated MECOM knockdown

Human ESC lines were used in accordance with the UK Stem Cell Bank Steering Committee guidelines (Project Approvals SCS11-51 and SCSC17-26). H9 hESC were differentiated to human embryonic stem cell-derived EC (hESC-EC) as previously described.^31,32^ Small interfering RNA (siRNA)-mediated knockdown of MECOM was performed using Day 7 hESC-EC using predesigned siRNA at a final concentration of 5 nM (Supplementary material online, *Table S2*). After 6 h, transfection media was replaced with EGM-2 media supplemented with 1% human AB serum and 50 ng/mL VEGF-A. At Day 10, CD144+ hESC-EC were isolated by magnetic activated cell sorting and cell pellets stored at −80°C for subsequent isolation of RNA and protein.

### 2.6 Bulk RNA-sequencing analysis

RNA was isolated from Day 10 CD144+ hESC-EC previously subjected to either transfection with siRNA targeting MECOM (siRNA 1) or control siRNA (*n* = 4 biological replicates). Illumina strand-specific RNA sequencing libraries with PolyA selection were prepared by GeneWiz (New Jersey, USA) and sequenced using the Illumina NovaSeq sequencer to achieve a read depth of 20 million paired end reads per sample.

Reads from each sample were mapped and quantified using RSEM^33^ (v1.3.0, –bowtie2) and the GENCODE v38 primary assembly transcriptome. Genes with an average FPKM >1 in one or the other experimental group were considered to be expressed. To identify DEGs, tximport^34^ (v1.22.0) was used to supply DESeq2^35^ (v1.34.0) with the isoform read counts from RSEM before using the default DESeq2 method (Wald test) to obtain gene-level *P*-values and fold changes between experimental conditions. Those genes with an absolute fold change value >1.5 (absolute Log2FC value >0.584) and adjusted *P*-value of <0.05 were considered differentially expressed. Over-represented KEGG terms amongst siMECOM-up-regulated genes were identified using clusterProfiler^36^ (v4.2.0) and Benjamini–Hochberg multiple hypothesis correction (*P* < 0.05).

All further experimental and analysis details are included in the Supplementary material online, *Methods*.

## 3. Results

### 3.1 Identification of distinct cardiac endothelial populations

**Figure 1:**
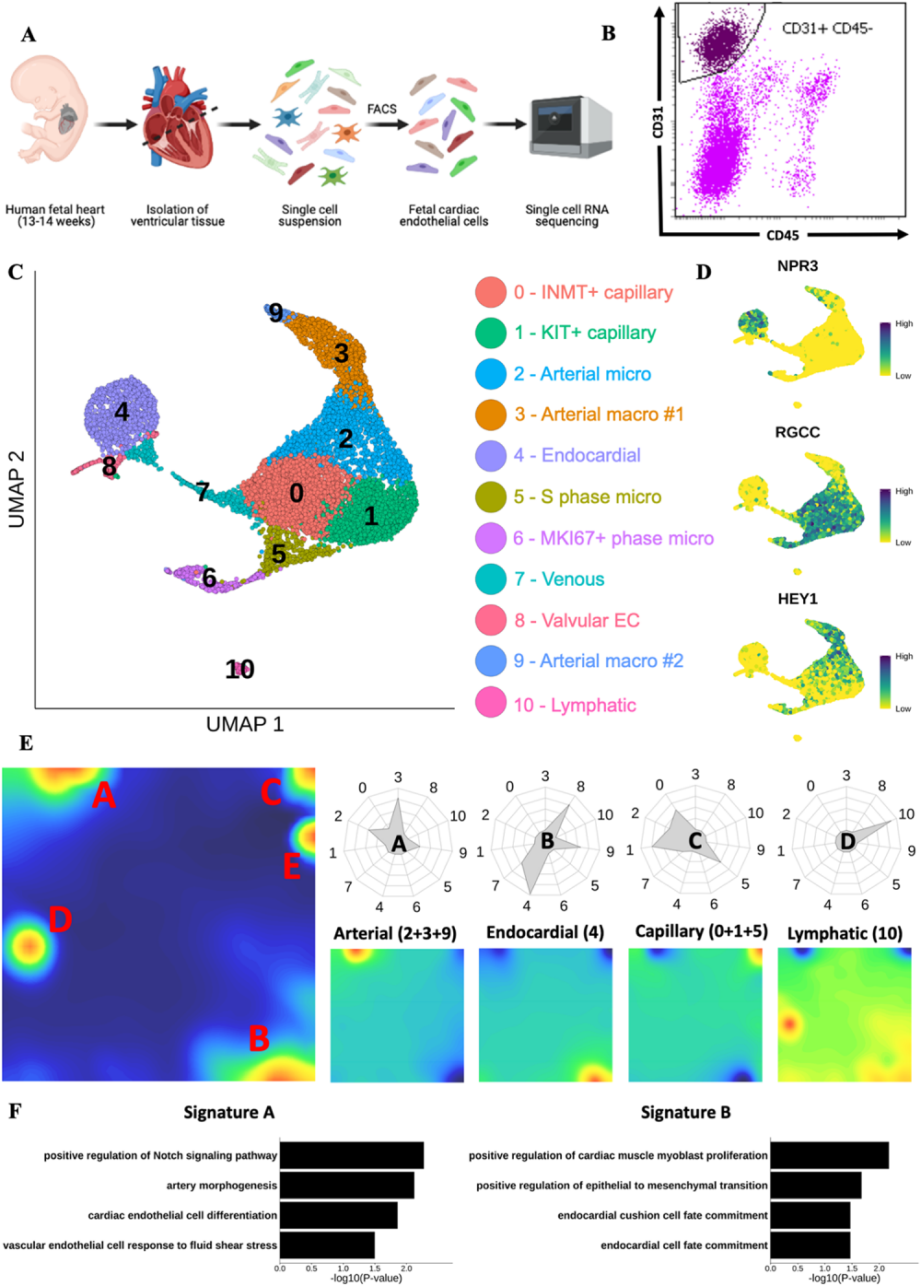
Mapping the human foetal heart endothelium using scRNA-seq. (*A*) Schematic of experimental design for mapping the human foetal heart endothelium using 10× scRNA-seq. (*B*) Representative FACS gating strategy used to isolate viable CD31+ CD45− ECs. (*C*) UMAP visualization of clusters identified in scRNA-seq data from cardiac ECs isolated from human foetal heart (*n* =2). (*D*) Feature plots showing expression of key marker genes defining distinct endothelial populations. (*E*) Metagene analysis of foetal heart scRNA-seq data visualized in self-organized maps for total dataset (left) and subpopulations of EC (right). Radar plots show enrichment of each metagene signature in individual clusters. (*F*) GO term enrichment analysis conducted using genes from metagenes’ signatures A (left) and B (right).

scRNA-seq was performed on CD31+ CD45− cardiac ECs isolated by FACS from ventricular tissue obtained from two human foetuses at 13 and 14 weeks of gestation (*Figure 1A and B*). At this developmental stage all major structures in the heart have formed, including the coronary vasculature. However, studies from equivalent timepoints in murine development (E15.5–E17.5) have revealed extensive remodelling occurring within the established coronary vasculature producing a mature vascular bed containing heterogeneous EC populations.^6,9,37^ Following quality control, unsupervised clustering, and UMAP visualization of transcriptomic data from 10 267 cells, 11 distinct clusters (numbered 0–10) were revealed, each with expression of typical pan-EC markers (*Figure 1C* and Supplementary material online, *Figure S1A*). Leukocyte marker *PTPRC* and fibroblast/smooth muscle cell markers *ACTA2* and *MYH11* demonstrated negligible expression across all clusters (Supplementary material online, *Figure S1A*). Annotating cells by sample demonstrated successful integration of datasets, with each cluster containing cells from both samples (Supplementary material online, *Figure S1B*).

Expression of *NPR3*, a known endocardial marker, was localized to cluster 4 (*Figure 1D* and Supplementary material online, *Figure S1C*).^38^ Clusters 0, 1, 2, 5, and 6 were defined by expression of capillary marker *RGCC*,^39^ whilst arterial marker, *HEY1,*^40^ was expressed predominantly in clusters 2, 3, and 9 (*Figure 1D* and Supplementary material online, *Figure S1C*). Metagene analysis revealed five major signatures (A–E) indicating five key populations within the data (*Figure 1E* and Supplementary material online, *File S1*). Signature A was enriched in clusters 2, 3, and 9, and included genes involved in arterial EC function, such as *JAG1, DLL4*, and *HEY1* (*Figure 1E*). Furthermore, Gene Ontology (GO) term enrichment analysis using signature A genes identified ‘artery morphogenesis’ and ‘positive regulation of Notch signalling pathway’ as significantly enriched terms (*Figure 1F*). Notch signalling is known to be required for arterial EC specification.^41^

**Figure 2:**
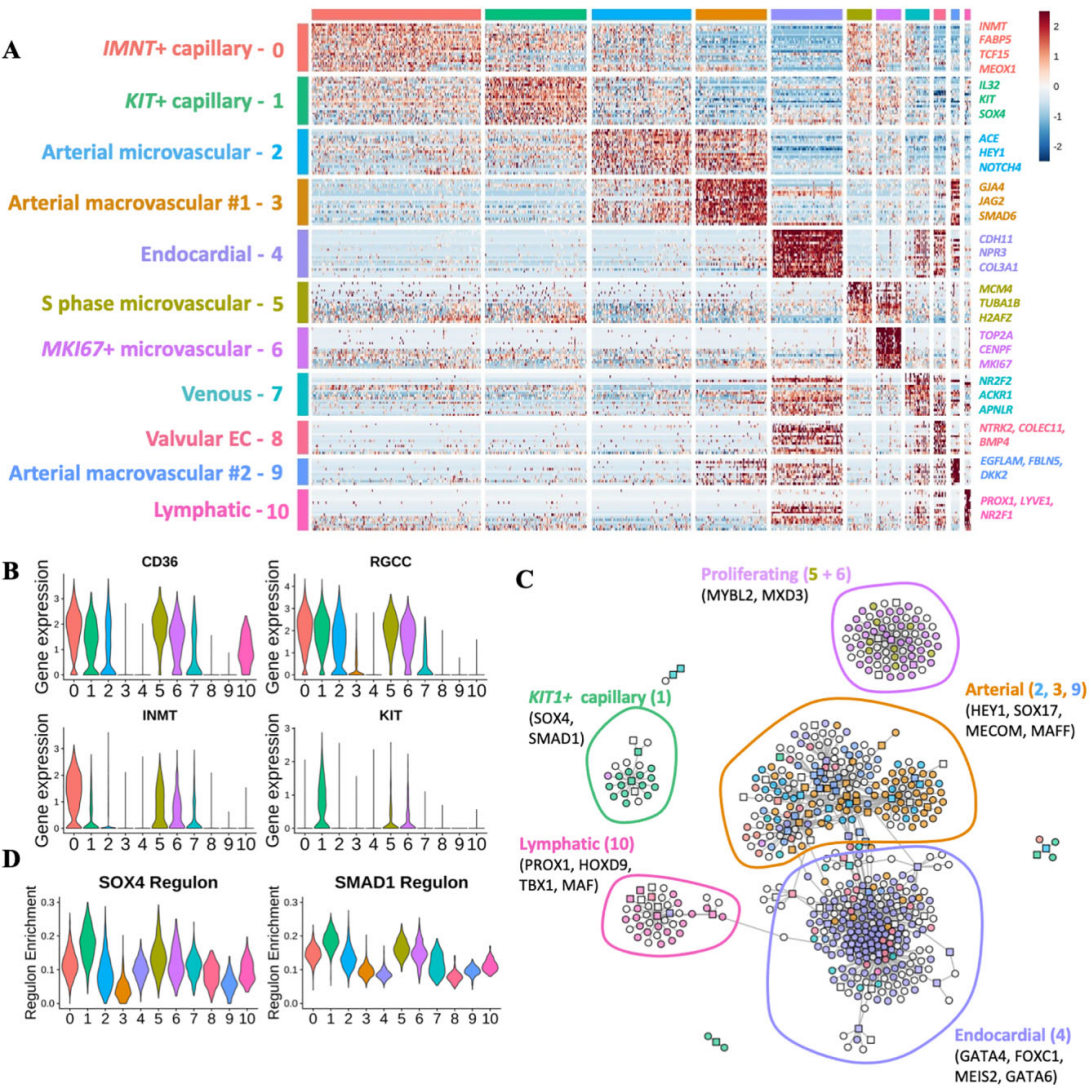
GRN analysis of human foetal heart endothelium. (*A*) Heatmap of differentially expressed cluster genes: expression of top 20 DEGs for each cluster identified in the complete dataset. Genes were grouped according to the cluster in which they were differentially expressed. (*B*) Violin plots: expression of *CD36, RGCC, INMT*, and *KIT* across clusters identified in foetal heart EC dataset. (*C*) GRN constructed using SCENIC analysis. TFs and target genes shown as squares or circles, respectively. Genes are coloured based on the cluster in which they were differentially expressed. White nodes represent gene targets that were not differentially expressed. (*D*) Violin plots showing enrichment/AUC score of *SOX4* and *SMAD1* regulons across identified clusters.

Signature B was predominantly enriched in Cluster 4 and contained known endocardial markers *CDH11* and *NPR3* (*Figure 1E and F*).^38,42^ Lower levels of signature B enrichment were also observed in Clusters 7, 8, and 9 (*Figure 1E*). Clusters 0, 1, and 5 were enriched for signature C, which included capillary EC marker genes, such as *CA4* and *RGCC* (*Figure 1E* and Supplementary material online, *Figure S1D*).^39,43^ Cluster 10 was enriched for signature D, for which GO term analysis returned terms relating to lymphatic EC (LEC) (*Figure 1E* and Supplementary material online, *Figure S1D*). LEC markers,^44^*LYVE1, FLT4, PROX1*, and *PDPN*, were differentially expressed in Cluster 10 (Supplementary material online, *Figure S1G*). Signature E was selective for Cluster 6 with GO term analysis identifying enriched terms relating to proliferation (Supplementary material online, *Figure S1E*). In line with this, categorizing cells according to their predicted cell cycle phase revealed that 77% of cells in Cluster 6 were predicted to be in the G2M phase of rapid growth, whereas 95% of cells in cluster 5 were in S phase (Supplementary material online, *Figure S1F*). The remaining cells in the dataset were predominantly in the G1 phase (79%), with only a small proportion predicted to be in G2M (4%) and S phases (17%) (Supplementary material online, *Figure S1F*). For clusters not associated with a metagene signature, analysis of DEGs revealed enrichment of *NR2F2* and *ACKR1* in Cluster 7, suggesting a venous/venular EC identity (*Figure 2A* and Supplementary material online, *Figure S1G*).^45,46^ A valvular identity of Cluster 8 was supported by its differential expression of *NFATC1* and *BMP4*, both with known roles in valvulogenesis (Supplementary material online, *Figure S1G*).^47,48^ Collectively, these analyses demonstrate that each major subtype of EC within the heart (endocardial, venous, lymphatic, capillary, arterial, proliferating, and valvular EC) is represented by one or more of the identified 10 clusters.

### 3.2 Gene regulatory network analysis of foetal cardiac ECs

Global differential gene expression analysis revealed markers for subpopulations of arterial and capillary cardiac EC (*Figure 2A* and Supplementary material online, *File S2*). Notably, Cluster 2, an arterial EC population, is more closely correlated with capillary EC clusters (0 and 1) than with the other two minor arterial clusters (3 and 9) (Supplementary material online, *Figure S2A*). Together with the co-expression of both arterial and capillary markers, this suggested that Cluster 2 represents an arterial microvascular population. In contrast, arterial clusters 3 and 9 DEGs associated with ECM organization (*FBLN5, ELN*, and *FBN1*) and shear stress (*KLF4)* suggesting a macrovascular identity (Supplementary material online, *Figure S2B* and *C*).

Expression of fatty acid translocase encoding *CD36* was absent from macrovascular and endocardial populations, corresponding with a previous report of microvasculature-restricted expression (*Figure 2A and B*).^49^ Differential gene expression analysis revealed heterogeneity between the two major capillary clusters, 0 and 1. Cluster 0 was defined by differential expression of amine methyltransferase encoding gene, *INMT*, whilst *KIT* expression was enriched in Cluster 1 (*Figure 2A and B*). Selective expression of *KIT* (also known as *C-KIT*) in Cluster 1 was accompanied by up-regulated expression of the TF *SMAD1* (Supplementary material online, *Figure S2D* and *E*).

Gene regulatory network (GRN) analysis was applied to identify gene modules, known as regulons, predicted to be controlled by an individual TF, giving insight into the likely transcriptional regulators of EC heterogeneity. Visualization of differentially expressed TFs and their predicted targets within the GRN largely recapitulated the data structure observed following unsupervised clustering (*Figure 2C*). Genes differentially expressed in LEC (Cluster 10) localized together in the GRN and included TFs, such as *PROX1*, a known master regulator of LEC identity,^50^ as well as *HOXD9* and *TBX1* (*Figure 2C*). A set of differentially expressed endocardial TFs (Cluster 4) was evident, including *GATA6, MEIS2*, and *FOXC1*, as well as *GATA4*, known to be implicated in endocardial cushion development.^51,52^*MECOM* and *MAFF* were located amongst known regulators of arterial EC specification, such as *HEY1* and *SOX17* (*Figure 2C*).^40,53^ A distinct cluster of genes differentially expressed in *KIT1*^+^ capillary EC (Cluster 1) included *SOX4* and *SMAD1* (*Figure 2C*). Enrichment of *SOX4* and *SMAD1* regulons was also observed in *KIT1*+ capillary EC (*Figure 2D*).

### 3.3 Trajectory analysis predicts an endocardial contribution to the developing coronary vasculature and potential regulators of subsequent arterial specification

Several recent studies using murine models of coronary vascular development have provided insight into the origin of the coronary endothelium and the dynamic changes that occur during its subsequent remodelling.^6,54^ Endocardial-derived vessels vascularize the heart from the inside-out contributing to vessels of the interventricular septum and inner myocardial wall.^1,55,56^ Conversely, SV-derived vessels populate the outer ventricular free walls of the heart from the outside-in.^38,55,56^ Following the formation of the primitive coronary vascular plexus, EC undergo further remodelling to form a functional network of veins, arteries, and capillaries.^9^

**Figure 3:**
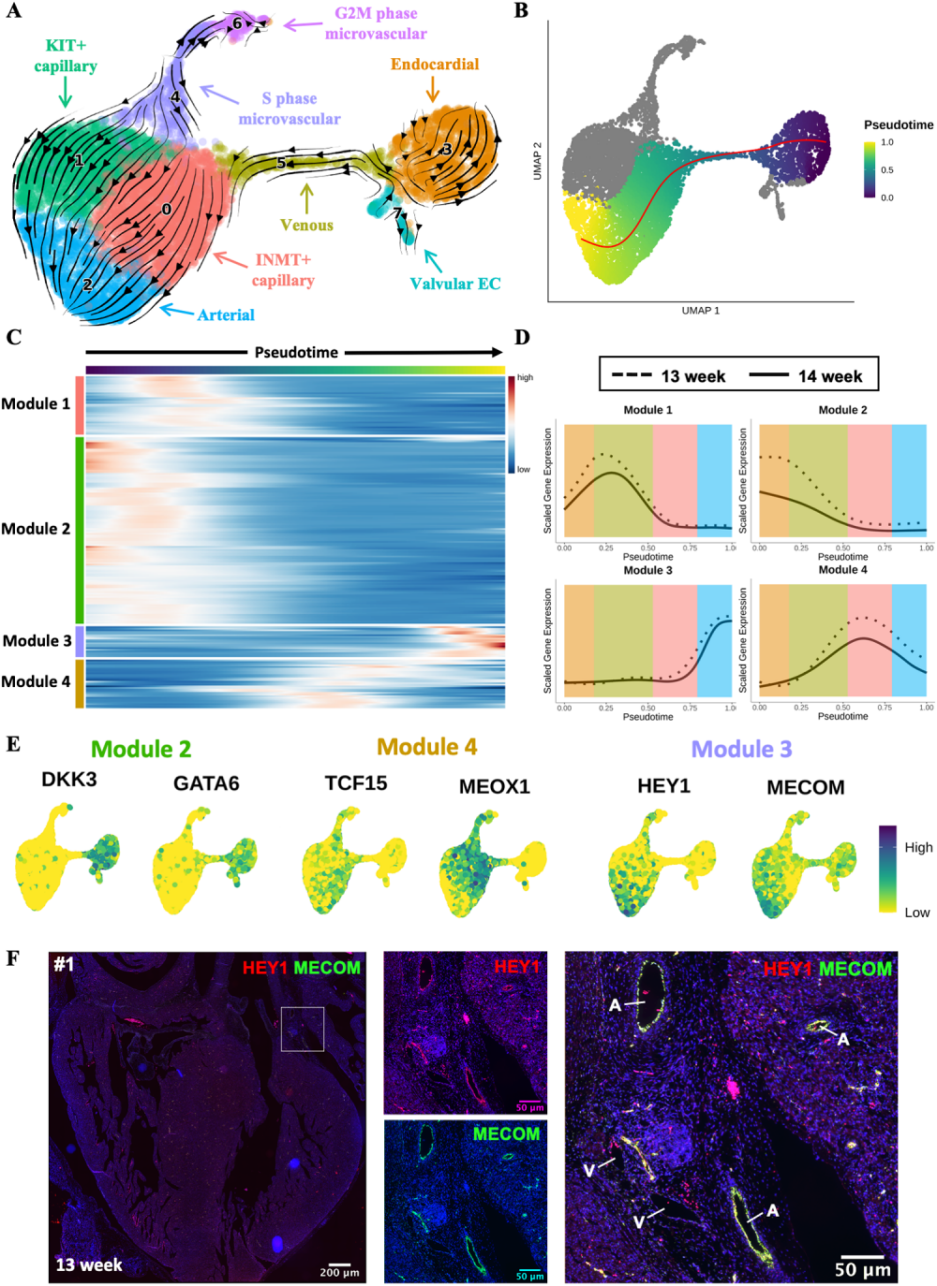
Trajectory inference analysis of developing cardiac endothelium. (*A*) RNA velocity analysis of microvascular cardiac endothelium. The RNA velocity field shown superimposed onto a UMAP visualization of microvascular cardiac ECs. (*B*) Slingshot trajectory demonstrating pseudotemporal cellular dynamics. (*C*) Heatmap of 200 genes found to be most differentially expressed across pseudotime of trajectory from (*B*). Genes grouped into modules by *k*-means clustering (*k* = 4). (*D*) Smoothing spline curves show average sample scaled gene expression for genes within modules identified in (*A*). (*E*) Feature plots showing expression of selected genes from Modules 2 (*DKK3* and *GATA6*), 3 (*MECOM* and *HEY1*), or 4 (*TCF15* and *MEOX1*). (*F*) ISH validation of co-expression of *MECOM* (green) with *HEY1* (red) in arterial EC of a 13-week human foetal heart (sample #1). See Supplementary material online, *Figure S5A* for samples #2, #3, and #4. A, artery; V, vein.

We used trajectory inference methods to determine whether these processes could be identified during human cardiac development and to characterize their accompanying transcriptional changes. Given that these dynamic changes are known to originate from microvascular EC, we excluded the two previously identified arterial macrovascular clusters (Clusters 3 and 9) from the dataset and performed secondary clustering of the remaining cells (*Figure 3A*). The distinct LEC cluster was also excluded prior to re-clustering. The same marker genes used for annotating the complete dataset were used for the annotation of re-clustered data (Supplementary material online, *Figure S3A*).

RNA velocity analysis,^28^ which utilizes the ratio of spliced to unspliced transcripts to infer the direction and magnitude of cellular transitions, was first used to gain an overview of the pseudotemporal dynamics of the foetal cardiac endothelium (*Figure 3A* and Supplementary material online, *Figure S3B*). We identified a proportion of the endocardial cluster with velocity vectors indicating a probable transition towards a venous identity (*Figure 3A* and Supplementary material online, *Figure S3B*). Evidence for this transition was further supported by venous EC-associated genes, such as *PLVAP* and *NR2F2*, having positive residuals/velocities in endocardial cells (Supplementary material online, *Figure S3C*). In turn, venous EC were subsequently predicted to transition to *INMT+* capillary EC. This predicted transition of endocardium to coronary vascular EC concurs with studies that identified the endocardium as a significant source of EC for the coronary vasculature. To further substantiate this finding, cells belonging to endocardial, venous, and *INMT+* capillary clusters were isolated *in silico* and re-clustered. UMAP visualization of re-clustered data revealed a comparable result to previous analysis with endocardial and *INMT*+ capillary populations connected by a *ACKR1+* venous population (Supplementary material online, *Figure S3D*). Additionally, the omission of cell cycle-related genes from clustering and visualization calculations generated a comparable finding, thus confirming localization of identified clusters was not confounded by cell cycle-related effects (Supplementary material online, *Figure S3E*). Velocity analysis also predicted a likely transition of both capillary EC populations towards an arterial EC fate (*Figure 3A* and Supplementary material online, *Figure S3B*). This is an agreement with previous reports of developmental arterial remodelling in mouse.^7–9^

In addition to the RNA velocity analysis, we also independently applied the trajectory inference tool Slingshot,^29^ which yielded a comparable interpretation (*Figure 3B*). Identification of the top 200 genes with most variable expression over pseudotime revealed four temporal patterns of expression, arranged in Modules 1–4 (*Figure 3C and D*). Average sample module gene expression was visualized over pseudotime to ensure concordant expression dynamics between individual samples. Module 2 genes were expressed early in pseudotime with their reduction in expression occurring in conjunction with the loss of endocardial identity (*Figure 3D*). These included known markers of endocardium, such as *CDH11* and *NPR3* as well as the TFs *DKK3* and *GATA6* (*Figure 3E* and Supplementary material online, *Figure S4A*). TFs *CEBPD* and *FOS* were identified in Module 1 along with *NR2F2*, a known regulator of venous EC specification^45^ (Supplementary material online, *Figure S4A*). Interestingly, despite not being identified within Module 1, expression of *BMP2* was found to increase within the pseudotime range corresponding to the predicted transitioning venous population (Supplementary material online, *Figure S4B*). As well as demonstrating enriched expression in venous EC in zebrafish,^57^ BMP2 has also recently been identified as positive regulator of endocardial to coronary vascular EC transition during murine cardiac development.^58^ Module 4 genes demonstrated peak expression within *IMNT*+ capillary EC and included TFs *TCF15* and *MEOX1* (*Figure 3E*). Expression of *DACH1* was found to peak within the *IMNT*^+^ capillary cluster before gradually decreasing again within the arterial population (Supplementary material online, *Figure S4C*). Previous studies have identified *Dach1* as a driver of developmental arterial remodelling in murine cardiac development.^8,9^

The predicted transition of capillary EC to arterial EC was defined by increased expression of Module 3 genes (*Figure 3C and D*). This included *HEY1*, known to mediate arterial EC specification.^40^ Interestingly, Module 3 also contained the TF *MECOM*, earlier predicted by GRN analysis to underlie arterial EC identity (*Figures 2C and 3C–E* and Supplementary material online, *Figure S4A*). Subsequent *in situ* hybridization (ISH) validation conducted across four independent foetal hearts (aged 13–14 weeks) demonstrated clear co-expression of *MECOM* with the arterial EC enriched TF *HEY1* within arterial vessels (*Figure 3F* and Supplementary material online, *Figure S5A* and *B*). Notably, a lack of *MECOM* expression was observed in vessels with venous morphology validating its arterial EC specificity (*Figure 3F* and Supplementary material online, *Figure S5A* and *B*). In addition, reanalysis of publicly available scRNA-seq data from healthy human foetal heart data from Suryawanshi *et al.*^12^ revealed *MECOM* expression to be enriched within a subset of the endocardial/endothelial population with minimal expression in other identified cell types (Supplementary material online, *Figure S6A–C*).

### 3.4 Comparison with murine coronary developmental gene expression reveals conserved markers of cardiac EC populations

Our current understanding of cardiac vascular development is derived predominantly from murine models. Consequently, we next compared the transcriptional profiles of developing foetal human and embryonic mouse cardiac EC. For this comparison, a publicly available mouse embryonic heart scRNA-seq dataset^15^ was used due to its good representation of cardiac EC and because its embryonic stage (E16.5) corresponded with the later developmental stage of our human foetal heart data (13–14 weeks).^37^

**Figure 4:**
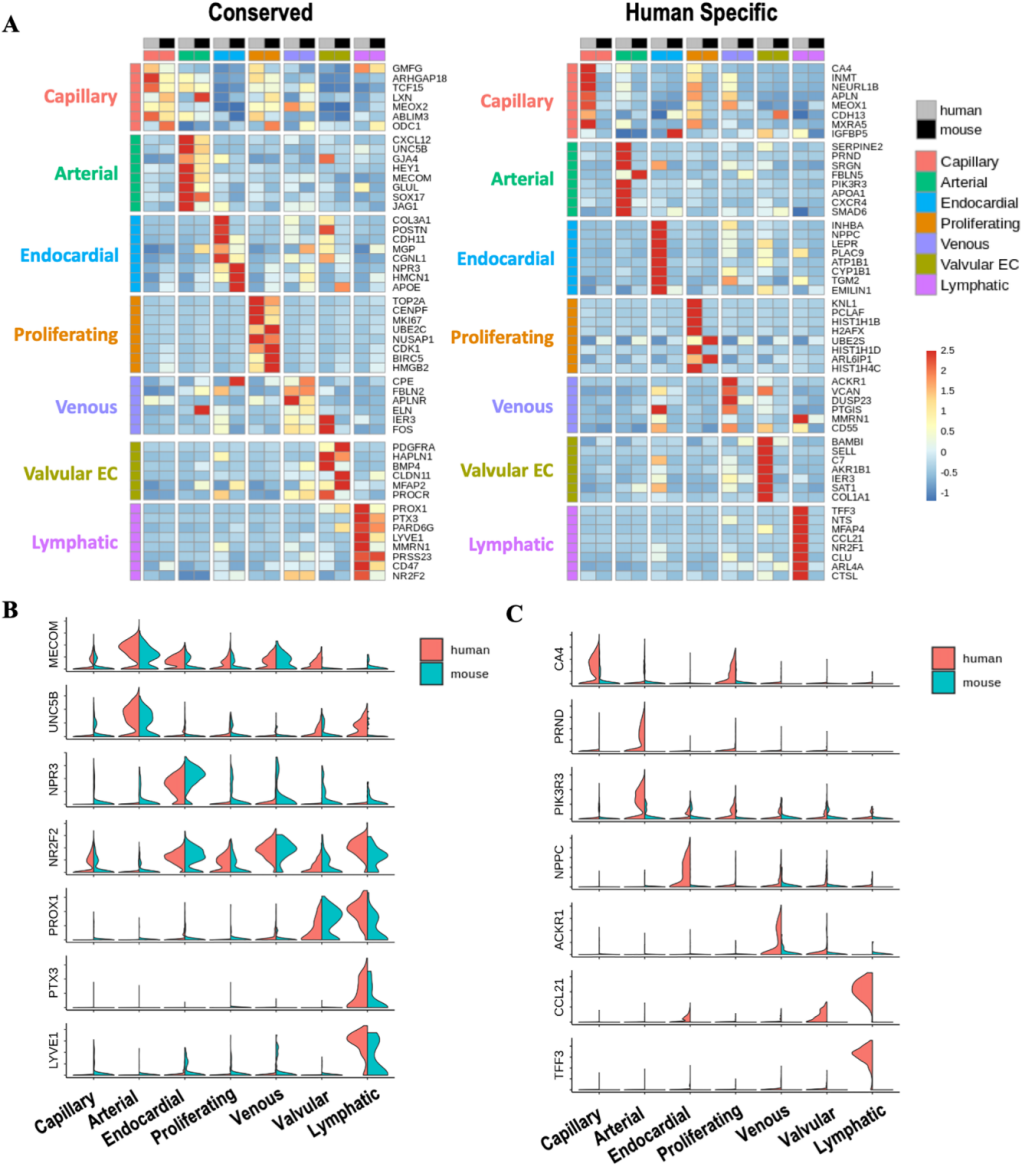
Comparison of the transcriptional profiles of human and mouse foetal cardiac EC populations. (*A*) Heatmaps showing expression of either conserved (left) or human-specific (right) markers for each subpopulation of foetal cardiac endothelium. (*B*) Expression of selected conserved markers in EC populations in human and mouse. (*C*) Expression of markers identified as being human-specific.

Dimensionality reduction revealed successful integration of mouse and human cardiac EC (Supplementary material online, *Figure S7A*). Unlike our observation in the human heart, no distinct populations of *KIT+* or *INMT+* capillary populations were observed in the mouse data (Supplementary material online, *Figure S7B*). Clusters were therefore merged to represent the major subtypes of EC within the heart (endocardial, venous, lymphatic, capillary, arterial, proliferating, and valvular EC). Genes found to be amongst the most significantly differentially expressed in the same population in both human and mouse were classified as conserved markers (*Figure 4A*). *MECOM* and *UNC5B* were among genes with enriched arterial EC expression in both species (*Figure 4A and B*). In agreement with previous findings in mouse,^38^*NPR3* expression was highly specific to the endocardial population with minimal expression in valvular EC.^38^ A lack of clear conserved venous EC markers was observed, with partial overlap of DEG in some lymphatic, valvular, and endocardial populations (*Figure 4A and B*). Interestingly, whilst the known LEC TF, *PROX1*, was expressed in both valvular EC and LEC in both species, *PTX3* and a LEC marker, *LYVE1*, were found to be highly LEC specific (*Figure 4A and B*). Species-specific markers were also identified for each EC dataset (*Figure 4A* and Supplementary material online, *Figure S7C*). A human endocardial marker, *NPCC*, described elsewhere as specifically defining human foetal cardiac endocardium,^12^ was not enriched in the corresponding mouse population (*Figure 4C*).

### 3.5 MECOM is required in arterial-like hESC-EC to suppress non-arterial gene expression

Given the *in-silico* predictions of a role for *MECOM* in arterial fate and enriched *MECOM* expression in arterial EC for both human and mouse, suggesting an evolutionarily conserved role in arterial EC, we sought to validate its role in determining human arterial EC identity. Our previous scRNA-seq-based characterization of our 8 day hESC-EC differentiation protocol demonstrated its suitability as an *in vitro* model of human EC development.^32^ Additionally, we determined that after acquisition of an early EC identity by Day 6, hESC-EC assume a clear arterial-like EC transcriptional signature by Day 8, characterized by expression of arterial markers, such as *SOX17* and *DLL4* (Supplementary material online, *Figure S8A*). Expression of venous (*NR2F2* and *EPHB4*) and lymphatic markers (*PROX1*) in hESC-EC was low by Day 8 of the differentiation (Supplementary material online, *Figure S8A*). Notably, in agreement with its arterial EC specificity, *MECOM* was specifically expressed in hESC-EC at Days 6 and 8 of differentiation (Supplementary material online, *Figure S8A*).

**Figure 5:**
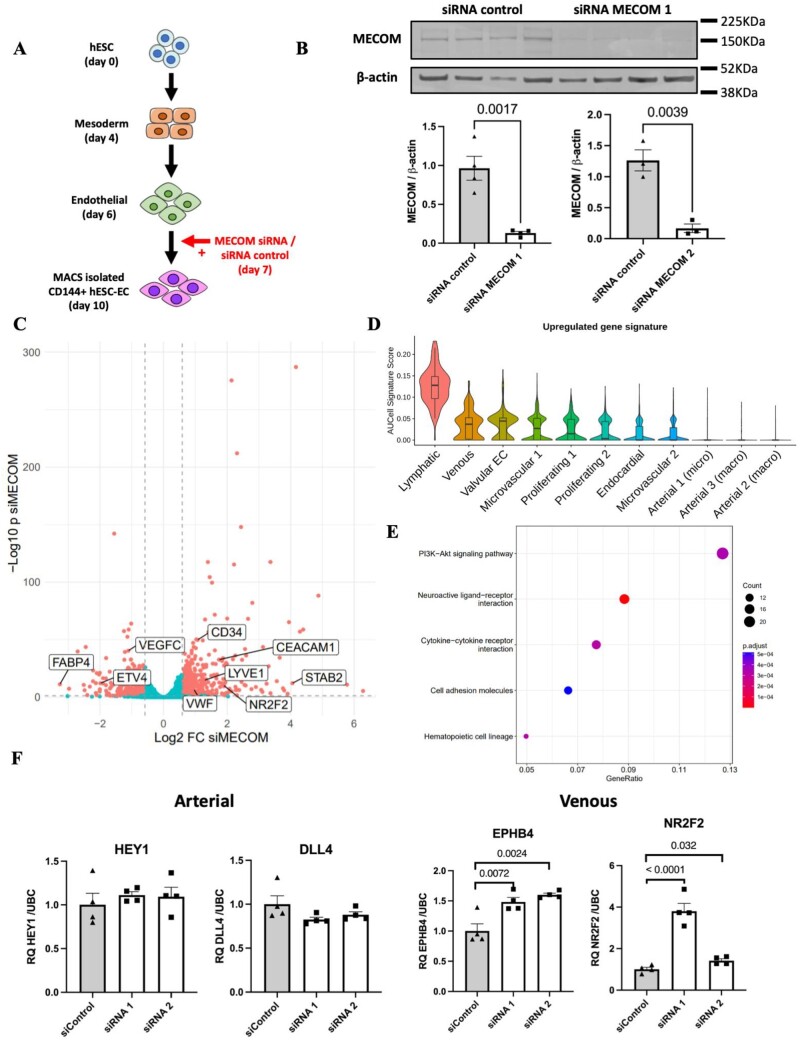
Knockdown of MECOM in hESC-EC. (*A*) Experimental design for siRNA-mediated knockdown of MECOM in hESC-EC. (*B*) Quantification of MECOM protein abundance following siRNA knockdown using siRNA MECOM 1 (*n* = 4 biological replicates) and siRNA MECOM 2 (*n* = 3 biological replicates). *P*-values were obtained using an unpaired *t*-test. (*C*) Volcano plot showing differential gene expression following MECOM siRNA-mediated knockdown in hESC-EC (*n* = 4 biological replicates). *P*-values calculated using the Wald test. (*D*) Up-regulated gene signature score applied across identified clusters in foetal heart EC scRNA-seq dataset. Up-regulated gene signature constructed using top 20 genes found to be significantly up-regulated following MECOM knockdown in hESC-EC. (*E*) KEGG pathway enrichment analysis conducted using significantly up-regulated genes following MECOM knockdown. (*F*) qRT–PCR quantification of known markers of arterial (*DLL4* and *HEY1*) and venous (*EPHB4* and *NR2F2*) EC after MECOM siRNA knockdown (*n* = 4 biological replicates). *P*-values were calculated using a one-way ANOVA followed by Dunnett’s *post-hoc* multiple comparison test. Graphs in (*B*) and (*F*) correspond to mean ± standard error of the mean.

Using hESC-EC as a developmental model for arterial EC specification, we next determined whether siRNA-mediated MECOM knockdown in hESC-EC resulted in changes to their arteriovenous identity (*Figure 5A*). Significant knockdown (>50%) of MECOM was observed at the RNA and protein level in hESC-EC 72 h after siRNA transfection (*Figure 5B* and Supplementary material online, *Figure S8B* and *C*). Bulk RNA-sequencing analysis revealed a distinct transcriptional profile for hESC-EC following MECOM knockdown compared to that of control hESC-EC (Supplementary material online, *Figure S8D*). Differential gene expression analysis demonstrated a reduction of MECOM resulted in a global increase in expression of non-arterial markers, including *NR2F2*^45^ (Log_2_FC = 1.89) and *VWF*^59^ (Log_2_FC = 0.95) known to be enriched in venous EC (*Figure 5C* and Supplementary material online, *File S3*). Notably, known arterial markers including *HEY1* and *DLL4* were found not to be significantly down-regulated in response to MECOM knockdown. In addition to the up-regulation of known venous markers, several genes with previously reported differential expression in LEC including *LYVE1*^44^ (Log_2_FC = 1.29), *STAB2*^60^ (Log_2_FC = 4.05), and *CEACAM1*^61^ (Log_2_FC = 1.74) were also found to be significantly up-regulated (*Figure 5C*). However, expression of key LEC TF *PROX1* was not detected in either condition.

Application of an up-regulated gene expression signature (constructed using the top 20 up-regulated genes following MECOM KD in hESC-EC) to our foetal cardiac EC scRNA-seq dataset revealed the lowest level of signature enrichment in arterial populations, with highest levels of enrichment observed in lymphatic and venous clusters (*Figure 5D*). KEGG pathway enrichment analysis conducted using genes significantly up-regulated following MECOM KD identified enrichment of genes belonging to the PI3K-AKT signalling pathway, reported to play a role in venous EC specification^62,63^ (*Figure 5E*). qRT–PCR validation aligned with bulk RNA-seq findings demonstrating knockdown of MECOM resulted in significant up-regulation of venous EC markers (*NR2F2* and *EPHB4*) whilst arterial (*HEY1, DLL4, JAG1*, and *JAG2*) markers remained unchanged (*Figure 5F* and Supplementary material online, *Figure S8E*). Importantly, reduction of MECOM did not result in altered expression of the pan-endothelial marker, *CDH5*, suggesting that the changes observed in arteriovenous marker expression are not due to a loss of general EC identity (Supplementary material online, *Figure S8E*).

## 4. Discussion

In this study, we comprehensively mapped the transcriptional landscape of the developing human foetal heart endothelium using scRNA-seq. Isolation of foetal cardiac EC by FACS prior to performing high-throughput scRNA-seq empowered this study to identify the full extent of EC heterogeneity. This included identifying distinct endocardial, valvular, venous, capillary, and arterial EC populations each expressing a separate transcriptional signature.

GRN analysis identified the TFs most likely responsible for establishing the observed EC heterogeneity. Application of trajectory inference methods to microvascular ECs was used to map the cellular dynamics accompanying coronary vascular EC development. This revealed a small proportion of endocardial cells that appeared to transition to a vascular EC identity *via* a venous EC population. In addition, capillary EC was predicted to be undergoing specification to assume an arterial EC identity, defined by increasing expression of the TF, *MECOM*. Comparison of our human foetal heart EC data with E16.5 murine cardiac EC scRNA-seq data demonstrated the existence of several conserved, as well as species-specific, markers for each of the major cardiac EC populations. This included identifying *NPR3* and *MECOM* as conserved markers of endocardial and arterial populations, respectively. Finally, we demonstrated that loss of MECOM in arterial-like hESC-EC resulted in a global increased expression of non-arterial markers, suggesting a function to maintain identity in arterial EC.

In contrast to the capillary EC cluster defined by differential expression of methyltransferase-encoding gene, *INMT*, GRN analysis revealed several regulons enriched within the *KIT*+ capillary population. This included the *SMAD1* regulon. BMP/SMAD1 signalling has been demonstrated to promote angiogenesis whilst KIT/C-KIT has been shown to mediate neovascularization in retinal microvascular ECs in response to hypoxia.^64,65^ This suggests that *SMAD1* may mediate angiogenesis within hypoxic regions in the developing heart wall, although this will require further investigation to verify. The existence of two capillary populations with distinct transcriptional signatures, including the differential expression of *KIT* and *INMT*, was recently confirmed in an independent study from Phansalkar *et al.*,^19^ which performed low-throughput scRNA-seq on EC isolated from 11-, 14-, and 22-week human foetal hearts.

Our *in-silico* findings suggested a transition of endocardium to coronary vascular endothelium. This is consistent with previous findings from murine lineage-tracing studies, in which a proportion of the endocardium gives rise to coronary vascular EC *via* angiogenic sprouting.^1^ A second method of endocardial-derived coronary vessel formation during was also proposed to occur at the murine perinatal stage and involve the formation of new coronary vessels by the segregation of endocardial trabeculae protruding into the myocardium during compaction.^66^ However, this model has recently been challenged by Lu *et al.*,^67^ which concluded that formation of new coronary vessels during the perinatal stage is instead due to angiogenic expansion of the pre-existing coronary plexus.

Although our trajectory analysis indicated the transition of endocardium to coronary vasculature occurs *via* a venous EC population, the arteriovenous identity of cells undergoing this process has not previously been explored. Whilst studies in mouse have demonstrated a significant proportion of coronary vascular EC to be derived from venous cells of the SV, this is thought to occur much earlier in cardiac development than the comparative gestational age of the human foetal samples used in this analysis.^2,38^ However, the observed enrichment of *BMP2* expression within the identified venous cluster aligns closely with recent scRNA-seq evidence from D’Amato *et al.*^58^ identifying *Bmp2* as a marker of the transitioning endocardial population in E12 mouse embryos. Additionally, enriched venous expression of bmp2 has previously been described in zebrafish,^57^ thus, further indicating the identified venous cluster may represent a transitioning endocardial-derived population.

Trajectory inference analysis also revealed subsequent arterial specification of capillary EC. This predicted cellular transition in the human foetal heart was also recently identified by Phansalkar *et al.*,^19^ thus, collectively providing human relevance to current understanding of coronary artery development derived from murine studies.^7–9^ However, in addition to confirming the up-regulation of known mediators of arterial specification, such as *HEY1*^40^ and *SOX17*,^53^*MECOM* was also identified as having a role in the establishment of an arterial EC identity. Furthermore, enriched arterial expression of MECOM was also observed in coronary EC from E16.5 mouse hearts, suggesting an evolutionarily conserved function.

The localization of *MECOM* in the developing human heart was validated using ISH methods and its function in arterial EC identity demonstrated by siRNA-mediated knockdown in arterial-like hESC-EC. Previous work from Li *et al.*^68^ demonstrated that MECOM acts upstream of Notch signalling during zebrafish nephrogenesis. Given the importance of Notch signalling in arterial EC specification, this suggested that MECOM may alter arteriovenous identity by regulating Notch signalling. Whilst reduction in MECOM expression in hESC-EC did not alter the expression of arterial EC markers, including those belonging to the Notch pathway, a global increase in the expression of non-arterial-enriched genes was observed. This included the TF, *NR2F2*, which is known to establish venous identity, in part *via* repression of Notch signalling.^45^ Although a subset of genes up-regulated following MECOM knockdown have been reported to be differentially expressed in LEC, the absence of increased *PROX1* expression indicated the reduction in MECOM does not specify EC towards a lymphatic identity.

Collectively, these findings suggest that MECOM may be required to supress non-arterial gene expression during arterial EC specification. Goyama *et al.*^69^ previously demonstrated that loss of MECOM within Tie2+ cells results in severe vascular abnormalities leading to embryonic lethality in mouse between E13.5 and E16.5. However, considering our described findings, further investigation is required to characterize *MECOM* expression across the murine embryonic and adult coronary vascular endothelium, as well as to evaluate the resultant effect of EC-specific loss of MECOM on arteriovenous identity.

Previous studies from mouse have hypothesized that a venous identity is the default state for EC, with venous identity needing to be repressed via Notch signalling during arterial specification.^70,71^ Our finding that MECOM knockdown altered venous marker (*NR2F2* and *EPHB4*) expression, without changes to expression of Notch signalling genes, suggests that additional factors are required to supress venous identity during human arterial EC specification. However, further studies simultaneously targeting the expression of characterized arterial EC regulators is required to determine the position of MECOM within the hierarchal network of arteriovenous regulators. Previous findings in mouse demonstrated overexpression of arterial EC regulator Dach1 resulted in an increase in perfused arteries following myocardial infarction.^8^ Our finding from human data suggesting MECOM may function to maintain the transcriptional identity of arterial EC highlights it as a prime therapeutic candidate to drive arterialization in cardiovascular disease.

Although this study is the most comprehensive of its type to date, due to limited sample availability its data provides only a snapshot of a narrow developmental window (13–14 weeks). This limitation prevented the comparison of gene expression and cluster proportion between different gestational ages. Careful batch correction and visualization of gene expression dynamics across pseudotime for individual samples ensured findings from trajectory inference analysis were not biased by unequal representation of individual clusters. Whilst trajectory inference methods permit the dynamical changes to be characterized within individual datasets, inclusion of foetal samples from a wider range of gestational ages would provide a more comprehensive understanding of human coronary vascular development, especially at earlier stages.

In summary, we have used a high-throughput scRNA-seq platform to comprehensively map the transcriptional landscape of the human foetal heart endothelium at 13–14 weeks. This study complements studies using murine models of cardiovascular development by providing novel insight into EC heterogeneity within the developing human heart, as well as the dynamical changes accompanying coronary vasculature formation. In addition to helping understand the mechanisms giving rise to congenital coronary vascular abnormalities, this information may prove valuable in future strategies to guide coronary vascular formation for the treatment of coronary vascular disease.

## Supplementary material

Supplementary material is available at *Cardiovascular Research* online.

### Authors’ contributions

I.R.M., J.C.M., N.S., M.B., and A.H.B. were involved in the design of the described study. I.R.M. and R.D. carried out foetal tissue collection and sample processing. Bioinformatic analysis was performed by I.R.M. and M.B. *In-vitro* experiments, qRT–PCR, and western blotting analysis were conducted by I.R.M., R.P., and A.B. A.H.B., C.P.P., and M.B. supervised the research. A.H.B. secured research funding. I.R.M., M.B., N.C.H., J.C.M., P.R.R., C.P.P., N.S., M.B., and A.H.B. were involved in interpreting bioinformatics data. I.R.M. and A.H.B. wrote the manuscript with input from all authors. All authors discussed the data and edited the manuscript.

## Supplementary Material

cvac023_Supplementary_Data

## Data Availability

RNA-sequencing data used in this study are accessible from the Gene Expression Omnibus (accession number: GSE195911). Endogenous blood vessel formation in the adult heart following myocardial infarction is insufficient to support adequate survival of the remaining myocardium, often ultimately leading to heart failure. Improved understanding of the mechanisms regulating human coronary vessel formation is required to inform therapeutic strategies to reactivate developmental pathways promoting therapeutic angiogenesis in patients. We applied scRNA-seq to map the transcriptome of the endothelium of the developing human heart. We identified novel transcriptional signatures underlying the cellular heterogeneity and dynamic changes occurring within the developing cardiac endothelium. This included identifying and validating MECOM as a novel regulator of arterial EC identity, which may serve as a target for therapeutic neovascularization.

## Notes

This manuscript was handled by Professor Thomas Thum.
