# Supplementary material for: Mapping the developing human cardiac endothelium at single-cell resolution identifies MECOM as a regulator of arteriovenous gene expression
Source: Cardiovasc Res. 2022 Feb 25;118(14):2960–72. doi: 10.1093/cvr/cvac023 (PMC9648824; doi:10.1093/cvr/cvac023)

**Supplementary Methods**

**Isolation of endothelial cells from fetal cardiac tissue.**

Cardiac endothelial cells were isolated from human fetal heart tissue using a method adapted from van Beijnum et al^1^. Fetal heart tissue was kept on ice prior to digestion to minimise RNA degradation. Ventricular tissue was finely minced prior incubating with continuous agitation at 37 °C in a digestion solution consisting of 9 ml 0.1 % collagenase II (Thermo Fisher, Paisley, UK) and 1 ml dispase (2.5U/ml) (Thermo Fisher). After 20 minutes incubation, 75 µl of 1 mg/ml DNaseI (Roche, Basel, Switzerland) was added prior to incubation for a further 15 minutes. Digestion was then quenched by placing the sample on ice and adding 5 ml cold RPMI with 10 % FCS (Life Technologies, Paisley, UK). Undigested clumps of tissue were then excluded by passing the sample through a 100 µm cell strainer and rinsing with a further 5 ml of cold RPMI + 10 % FCS. Cells were then collected by centrifugation (500 g for 5 minutes at room temperature) and 2 ml red blood cell lysis buffer (Thermo Fisher) added for 2 minutes at room temperature before neutralising with cold 15 ml RPMI + 0.1 % BSA. Cells were then pelleted by centrifugation (500 g for 5 minutes at 4 °C). Cells resuspended in RPMI + 0.1 % BSA were then stained on ice with APC anti-human CD31 and PE anti-human CD45 (Supplementary Table 1) for 45 minutes before resuspending again in RPMI + 0.1 % BSA. DAPI was added prior to sorting to allow exclusion of dead cells. CD31+ CD45- endothelial cells were isolated by fluorescence activated cell sorting (FACS) using the BD FACS Aria III Fusion cytometer (Becton Dickinson, Franklin Lakes, USA).

**Single-cell RNA sequencing library construction.**

Cell viability and concentration were determined by staining a 10 µl aliquot of FACS sorted endothelial cells 1:1 with trypan blue and loading onto a haemocytometer and counting manually. Viability of cells from both samples exceeded 85 %. 8,000 cells were loaded onto the 10X Chromium Controller (10X Genomics, Pleasanton, USA) according to the manufacturer’s protocol. Library construction was conducted using the Single Cell 3’ Reagent Kit (V3.1) in accordance with the manufacturer’s instructions (10X Genomics, Pleasanton, USA). In brief, following generation of the gel bead in emulsions (GEMs) and reverse transcription, cDNA was extracted from GEMs and amplified. cDNA clean-up was performed using a SPRIselect Reagent Kit (Beckman Coulter, Brea, USA). Indexed sequencing libraries were generated using the V3.1 Chromium Single-Cell 3' Library Kit (10X Genomics, Pleasanton, USA). The key steps in this protocol included enzymatic fragmentation, end-repair, A-tailing, adaptor ligation, ligation clean-up, sample index PCR, and PCR clean-up. Libraries were sequenced by Genewiz (USA) using the Illumina NovaSeq 6000 platform.

**Single-cell RNA sequencing data processing.**

Raw sequencing data was processed using the 10X CellRanger V3.1.0 pipeline (10X Genomics, Pleasanton, USA) to generate filtered and non-filtered count matrices. RNA-seq reads were aligned to the GRCh38-3.0.0 genome reference downloaded from 10X Genomics. In addition to the default cell calling method applied within the CellRanger pipeline, the DropUtils package was used with a FDR threshold of 0.01 to identify barcodes corresponding to cells *versus* those from empty droplets^2^. Only data from cells identified using both methods were used for downstream analysis. Quality control was performed using the R Scater package^3^. Cells with a total UMI count exceeding 3 median absolute deviations (MADs) from the median value or with total gene count lower than 2 MADs from the median value were removed from downstream analysis. In addition, cells with a high proportion of counts from mitochondrial genes (>3 MADs) were also excluded. Prior to merging individual datasets, data normalisation was performed using the MultiBatchNormalisation method^4^ to minimise batch effects between datasets. The top 2,000 most variable genes were subsequently identified using the standard Seurat FindVariableFeatures method. Mitochondrial and ribosomal genes were excluded from the list of variable genes. Normalised data was then scaled using the standard ScaleData function^5^ and principal component analysis conducted using the previously identified list of variable genes. Harmony batch correction was implemented to integrate both datasets, following the standard workflow^6^. Cells were clustered and visualised in two dimensions using Uniform Manifold Approximation and Projection (UMAP) following the standard Seurat workflow and utilising the corrected harmony reduction embedding values^5^. Fetal cardiac EC were assigned cell cycle stage using the list of cell cycle genes from Tirosh et al^7^ and implementation of the ‘CellCycleScoring’ function^5^. Differential gene expression analysis was performed using the standard FindMarkers method^5^. Significantly differentially expressed genes were determined using the Wilcoxon signed rank test with Bonferroni correction (adjusted p value < 0.05). A log_e_ fold change threshold of 0.3 was used when identifying differentially expressed genes (DEGs). Furthermore, DEGs were only considered further if expressed in more than 30 % of cells within their corresponding cluster. DEGs were then ranked based on their log fold change in expression. A complete list of DEGs is provided in Supplementary File 2. The AUCell package^8^ was applied to construct the upregulated gene signature using the top 20 most upregulated genes (ranked by log_2_ fold change in expression) in bulk RNA seq data of hESC-EC following MECOM knockdown. Publicly available scRNA-seq data from Suryawanshi et al^9^ was downloaded from the Gene Expression Omnibus and processed using the analysis workflow described above.

All raw sequencing data generated from this study has been deposited in the Gene Expression Omnibus (accession number GSE195911).

**Metagene signature analysis.**

The R package SCRAT v1.0.0 ^10^ was used to map the transcriptional signatures underlying the heterogeneity observed in the data. Normalised count data was used as the input data to construct self-organising maps (SOM) for the entire dataset and individual clusters. Enriched metagene signatures within individual cells were identified by applying a threshold retaining only metagene signatures with a signature score within the top 5% of signature scores (threshold score = max cell signature score x 0.95). Radar plots were constructed to visualise the proportion of cells within each cluster that exceeded the threshold for each metagene signature. GO term analysis was conducted using the R package topGO ^11^ using all detected genes as the background and the Fisher exact test with the "elim" algorithm to determine relevant significantly enriched GO terms.

**Gene regulatory network analysis.**

Gene regulatory network analysis performed using the standard R SCENIC (Single Cell rEgulatory Network Inference and Clustering) workflow ^8^. Briefly, the log normalised count matrix and accompanying cluster annotations were used as the input to GENIE3 ^12^ to infer potential transcription factor targets. A gene filter was first applied to remove genes with zero counts or those expressed in fewer than 10 cells. The Spearman’s rank correlation coefficient matrix for the input expression matrix was calculated using the default ‘runCorrelation’ command. Results from GENIE3 were then used to generate co-expression modules before performing cis-regulatory motif analysis for each transcription factor to identify predicted regulons ^8^. Motif enrichment analysis was performed using the two provided human RcisTarget databases ^8^ which provide rankings for motifs up to 500 bp upstream of the transcriptional start site (TSS) and for those in the 20 kbp region around the TSS. Regulons within cells were then scored using AUCell^8^. Motifs annotated to the corresponding transcription factor and with a normalised enrichment score (NES) greater than 3.0 were retained for downstream analysis. Regulon enrichment scores, represented by AUCell values, were visualised using violin plots for each of the previously identified clusters. The gene regulatory network was constructed using the R package iGraph including only differentially expressed transcription factors and their predicted targets with a Genie3 weighting exceeding 0.01.

**Trajectory inference analysis.**

Clusters corresponding to lymphatic (*PROX1^+^, LYVE1^+^)* and macrovascular (*FBLN5*^+^, *ELN*^+^) endothelial cells were removed from the dataset prior to reclustering and applying trajectory inference methods. RNA velocity analysis using the python package scVelo^13^ was applied using the stochastic model to determine the direction and magnitude of the inferred cellular dynamics. Identified trajectories were confirmed using the R package Slingshot^14^ following the standard pipeline and with the endocardial cluster assigned as the starting cluster in accordance with findings from RNA velocity analysis. Genes differentially expressed across pseudotime were then identified using the TradeSeq package using the standard workflow^15^. Briefly, a negative binomial generalised additive model (NB-GAM) was fitted for the 2,000 genes identified as having the most variable expression within the dataset. The optimal number of knots used when fitting the NB-GAM was determined as 7 by using the ‘Evaluate K’ function and selecting the number of knots after which the Akaike Information Criterion (AIC) value remained approximately constant ^15^. The 200 genes most differentially expressed across pseudotime were identified using the ‘associationTest’ function in which significantly differentially expressed genes (p<0.05) were then ranked using the Wald test statistic^15^.

**Multispecies comparison.**

Human fetal cardiac EC scRNA-seq data was compared to equivalent 10X scRNA-seq data from E16.5 mouse hearts published previously by Goodyer et al^16^. Following downloading count matrices from the Gene Expression Omnibus (GSE132658), mouse data was processed as described above to identify cell clusters visualised using in 2D using UMAP. Mouse EC were identified in silico using the SingleR annotation package^17^ following the standard pipeline, as well as by differential expression of endothelial markers including *Pecam1, Kdr,* and *Cdh5*. Mouse data was then filtered to exclude non-EC from downstream analysis. Human orthologs for mouse genes were identified using the BioMart package^18^ to allow marker gene expression comparison between species. Count data from mouse and human datasets were then processed in parallel to normalise gene expression and identify variable genes using the standard ‘NormalizeData’ and ‘FindVariableFeatures’ functions^5^. Individual datasets from human and mouse were then integrated using the standard Seurat integration method, using the first 30 dimensions from canonical correlation analysis (CCA) to identify integration anchors prior to running the ‘IntegrateData’ function^5^. Mouse EC populations equivalent to those identified above in human fetal EC data were classified using the ‘TranferData’ function and the human EC dataset as an integrated reference^5^.

The coefficient of variation (CV) was then calculated from normalised expression values for individual genes in human and mouse EC datasets, retaining only genes with a CV between the 5^th^ and 95^th^ percentiles. This step was necessary prior to differential gene expression analysis to exclude genes with low variability across all cells (housekeeper genes) as well as highly variable genes with sporadic expression within a low number of cells. Differentially expressed genes (DEGs) for each EC population in mouse and human datasets were then identified using the ‘FindAllMarkers’ function^5^ as described above. Marker genes were classified as conserved if found to be differentially expressed within the same EC population in both mouse and human datasets.

**Human embryonic stem cell (hESC) maintenance and endothelial differentiation.**

H9 hESC (passage number 38 - 47) were maintained in StemPro hESC SFM (Thermo Fisher) on vitronectin coated plates (human recombinant vitronectin; Thermo Fisher). Cells were mechanically passaged at ~90 % confluence at a ratio of 1:6 – 1:8 using the StemPro EZ passage tool (Life Technologies). hESC lines were used in accordance with the UK Stem Cell Bank Steering Committee guidelines (Project Approvals SCS11-51 and SCSC17-26). hESC were differentiated to endothelial cells as previously described^19, 20^. hESC at ~90 % confluence were dissociated to single cells using StemPro Accutase (Life Technologies) before resuspending in mTeSR1 media (Stemcell Technologies, Vancouver, Canada) supplemented with 10 μM Y27632 (Tocris, Bristol, UK). Cells were then replated in T25 flasks (12,000 cells/ cm^2^) coated 1 hour previously with fibronectin (5μg/cm^2^). 24 hours later (day 1) media was changed to N2B27 media (comprising of 25ml DMEM/F12/GlutaMAX medium + 25 ml Neurobasal medium + 250µl GlutaMAX (100X) + 1ml B27 supplement + 500µl N2 supplement + 0.5 mL β-mercaptoethanol; Thermo Fisher) supplemented with 7μM CHIR-99021 (Sigma, St. Louis, USA) and 25 ng/ml BMP4 (R&D Systems, Minneapolis, USA). Cells were then left for 3 days without media change. At days 4 and 5, media was changed for StemPro34 media supplemented with 200 ng/ml VEGF-A (R&D) and 2μM forskolin (Sigma). At day 6 cells were dissociated using 1X TrypLE Express (Life Technologies) and replated on uncoated flasks (40,000 cells/cm^2^) in EGM2 media (Lonza, Basel, Switzerland) containing 50 ng/ml VEGF-A and 1% human AB serum (Sigma).

**MECOM siRNA knockdown**

Small interfering RNA (siRNA) -mediated knockdown of MECOM in day 7 hESC-EC was performed using predesigned SilencerSelect siRNA (Thermo Fisher) at a final concentration of 5 nM (Supplementary Table 2). To reduce the likelihood of siRNA specific off-target effects, two independent siRNA were used in parallel to knockdown MECOM. siRNA transfection was performed by combining 2 ml of diluted Lipofectamine RNAiMAX reagent (12 μl in 2 ml Opti-MEM media) (Thermo Fisher) with 2 ml diluted siRNA (diluted in 2 ml Opti-MEM media to achieve a final concentration of 5 nM) and allowing complexes to form for 15 minutes at room temperature. Following washing cells with Opti-MEM media, siRNA-lipid complexes were added, and cells incubated at 37 °C / 5 % CO_2_. After 6 hours, transfection media was replaced with EGM-2 media supplemented with 1 % human AB serum and 50 ng/ml VEGF-A. Media was refreshed 48 hours later at day 9 before harvesting CD144+ cells at day 10 for RNA and protein. Day 10 cells were dissociated using 1X TrypLE Express and pelleted by centrifugation at 300 g for 3 minutes. Cells were washed once in DPBS-/- (minus calcium and magnesium) before resuspending in 80 μl MACS buffer (DPBS, 0.05 % BSA, 2 mM EDTA) and 20 μl CD144 MACS beads (Miltenyi Biotech, Bergisch Gladbach, Germany). Following staining at 4 °C for 15 minutes, CD144+ cells were isolated using MS columns (Miltenyi Biotech) according to manufacturer’s instructions.

**Quantitative real-time polymerase chain reaction (qRT-PCR)**

Total RNA was isolated from CD144+ hESC-EC using the miRNAeasy kit (Qiagen) following manufacturer’s instructions. Reverse transcription was then conducted with the Multiscribe Reverse Transcriptase kit (Thermo Fisher) using 400 ng of total RNA. Quantitative real-time polymerase chain reaction (qRT-PCR) was performed using predesigned TaqMan probes (Supplementary Table 3) and the QuantStudio 5 Real-time PCR system (Thermo Fisher) with a programme of 10 minutes at 95 °C followed by 40 cycles of 15 seconds at 95 °C and 60 seconds at 60 °C. Ubiquitin C was used as the housekeeper gene for normalisation (Thermo Fisher) and relative quantification calculated using the 2^-(ΔΔCT)^ (RQ) method.

**Western Blot**

CD144+ hESC-EC were pelleted (300 g, 3 minutes) and resuspended in cold RIPA buffer containing 1X cOmplete protease inhibitor cocktail (Sigma). After centrifugation at 10,000 g for 15 minutes at 4°C the supernatant was collected, and protein quantified using the Pierce BCA Protein Assay Kit (Life Technologies). Following adding 6X laemmli SDS reducing buffer (Thermo Fisher) and denaturing at 95 °C for 10 minutes, protein was separated on a 4-12 % Bolt Bis-Tris 1mm gel (Thermo Fisher) before transferring to a nitrocellulose membrane (Life Technologies). Membranes were blocked in 4 % milk for 1 hour at room temperature and incubated in primary antibody solution diluted overnight at 4 °C with continuous agitation (Supplementary Table 4). After washing 3 times in TBST, membranes were stained with secondary LiCor antibodies (Lincoln, USA) diluted in 4 % milk (Supplementary Table 4). Following 3 further washes in TBST, membranes were imaged using the LiCor CLx imaging system. Uncropped blot images are provided in Supplementary Figure 9.

**In-situ hybridisation validations**

Human fetal tissue from terminated pregnancies was obtained from the joint MRC/Wellcome Trust funded Human Developmental Biology Resource (HDBR, http://www.hdbr.org). All tissue was collected with appropriate maternal consent and approval from the London-Fulham NHS Health Authority Research Ethics Committee (Rec Reference 18/LO/0822). The HDBR centre is licensed by the UK Human Tissue Authority (license number 1220). Whole fetal heart samples from 13 and 14 post-conception weeks (PCW) were used for ISH validations. Whole fresh fetal hearts were fixed overnight at 4^o^C in 10% Formalin prior to paraffin embedding. 5 μM thick coronal paraffin sections were cut and consecutive sections were selected which captured both atrium and ventricles of the heart.

RNAscope in situ hybridization was performed using the Multiplex Fluorescent v2 Assay (Abingdon, United Kingdom) following the manufacturer’s instructions. Briefly, slides were baked for 1hr at 60oC, deparaffinized in a histoclear gradient and cleared in 100% ethanol. Sections were boiled in RNAscope Target Retrieval Reagent for 15 mins, followed by a 30min Protease pretreatment using RNAscope Protease Plus at 40^o^C to increase target accessibility. Probes for HEY1 (C1) and MECOM (C2) were used from the Bio-Techne Ltd (Abingdon, UK) Catalogue. The RNAScope in situ assay was also performed with a negative control probe, targeting the bacterial DapB gene, and a positive control probe for Cyclophilin B for each sample. The tissue sections were hybridised with the target probe mix for 2hrs in a humidified chamber at 40^o^C. The hybridized signals were then amplified by incubation with RNAscope Multiplex FL v2 Amp 1 for 30min at 40^o^C. For dual labelling sections were additionally incubated in RNAscope Multiplex FL v2 Amp 2 for 30min in a humidified chamber at 40^o^C. Sections were then incubated in in RNAscope Multiplex FL v2 HRP-C1 for 15min at 40^o^C prior to incubation with the appropriate Opal dye (Opal 650, 520 or 570 diluted 1:15000, Akoya Biosciences, Inc.) for 30min at 40^o^C and finally Incubated in RNAscope Multiplex FL v2 HRP blocker for 15min at 40^o^C. For dual staining, this procedure was repeated by incubating the sections with RNAscope Multiplex FL v2 HRP-C2 for 15min at 40^o^C, incubation with the appropriate Opal dye for 30min at 40^o^C and a 15min block at 40^o^C with RNAscope Multiplex FL v2 HRP block. Finally, the sections were counterstained with DAPI 30sec at RT, and mounted with ProLong Gold Antifade Mountant (ThermoFisher). Sections were imaged using a Zeiss Observer Fluorescence microscope with a x20 objective and images processed using Zen Blue (Pro) software.

**Supplementary References**

1. van Beijnum JR, Rousch M, Castermans K, van der Linden E, Griffioen AW. Isolation of endothelial cells from fresh tissues. *Nat Protoc* 2008;**3**:1085-1091.

2. Lun ATL, Riesenfeld S, Andrews T, Dao TP, Gomes T, Marioni JC, participants in the 1st Human Cell Atlas J. EmptyDrops: distinguishing cells from empty droplets in droplet-based single-cell RNA sequencing data. *Genome Biology* 2019;**20**:63.

3. McCarthy DJ, Campbell KR, Lun ATL, Wills QF. Scater: pre-processing, quality control, normalization and visualization of single-cell RNA-seq data in R. *Bioinformatics* 2017;**33**:1179-1186.

4. Haghverdi L, Lun ATL, Morgan MD, Marioni JC. Batch effects in single-cell RNA-sequencing data are corrected by matching mutual nearest neighbors. *Nature Biotechnology* 2018;**36**:421-427.

5. Stuart T, Butler A, Hoffman P, Hafemeister C, Papalexi E, Mauck WM, III, Hao Y, Stoeckius M, Smibert P, Satija R. Comprehensive Integration of Single-Cell Data. *Cell* 2019;**177**:1888-1902.e1821.

6. Korsunsky I, Millard N, Fan J, Slowikowski K, Zhang F, Wei K, Baglaenko Y, Brenner M, Loh P-r, Raychaudhuri S. Fast, sensitive and accurate integration of single-cell data with Harmony. *Nature Methods* 2019;**16**:1289-1296.

7. Tirosh I, Izar B, Prakadan SM, Wadsworth MH, 2nd, Treacy D, Trombetta JJ, Rotem A, Rodman C, Lian C, Murphy G, Fallahi-Sichani M, Dutton-Regester K, Lin JR, Cohen O, Shah P, Lu D, Genshaft AS, Hughes TK, Ziegler CG, Kazer SW, Gaillard A, Kolb KE, Villani AC, Johannessen CM, Andreev AY, Van Allen EM, Bertagnolli M, Sorger PK, Sullivan RJ, Flaherty KT, Frederick DT, Jané-Valbuena J, Yoon CH, Rozenblatt-Rosen O, Shalek AK, Regev A, Garraway LA. Dissecting the multicellular ecosystem of metastatic melanoma by single-cell RNA-seq. *Science* 2016;**352**:189-196.

8. Aibar S, Gonzalez-Blas CB, Moerman T, Huynh-Thu VA, Imrichova H, Hulselmans G, Rambow F, Marine JC, Geurts P, Aerts J, van den Oord J, Atak ZK, Wouters J, Aerts S. SCENIC: single-cell regulatory network inference and clustering. *Nat Methods* 2017;**14**:1083-1086.

9. Suryawanshi H, Clancy R, Morozov P, Halushka MK, Buyon JP, Tuschl T. Cell atlas of the foetal human heart and implications for autoimmune-mediated congenital heart block. *Cardiovasc Res* 2020;**116**:1446-1457.

10. Camp JG, Sekine K, Gerber T, Loeffler-Wirth H, Binder H, Gac M, Kanton S, Kageyama J, Damm G, Seehofer D, Belicova L, Bickle M, Barsacchi R, Okuda R, Yoshizawa E, Kimura M, Ayabe H, Taniguchi H, Takebe T, Treutlein B. Multilineage communication regulates human liver bud development from pluripotency. Nature. England, 2017:533-538.

11. A A, J R. *topGO: Enrichment Analysis for Gene Ontology* . R package version 2.34.0., 2018.

12. Huynh-Thu VA, Irrthum A, Wehenkel L, Geurts P. Inferring regulatory networks from expression data using tree-based methods. *PLoS One* 2010;**5**.

13. Bergen V, Lange M, Peidli S, Wolf FA, Theis FJ. Generalizing RNA velocity to transient cell states through dynamical modeling. *Nature Biotechnology* 2020;**38**:1408-1414.

14. Street K, Risso D, Fletcher RB, Das D, Ngai J, Yosef N, Purdom E, Dudoit S. Slingshot: cell lineage and pseudotime inference for single-cell transcriptomics. *BMC Genomics* 2018;**19**:477.

15. Van den Berge K, Roux de Bézieux H, Street K, Saelens W, Cannoodt R, Saeys Y, Dudoit S, Clement L. Trajectory-based differential expression analysis for single-cell sequencing data. *Nature Communications* 2020;**11**:1201.

16. Goodyer WR, Beyersdorf BM, Paik DT, Tian L, Li G, Buikema JW, Chirikian O, Choi S, Venkatraman S, Adams EL, Tessier-Lavigne M, Wu JC, Wu SM. Transcriptomic Profiling of the Developing Cardiac Conduction System at Single-Cell Resolution. *Circ Res* 2019;**125**:379-397.

17. Aran D, Looney AP, Liu L, Wu E, Fong V, Hsu A, Chak S, Naikawadi RP, Wolters PJ, Abate AR, Butte AJ, Bhattacharya M. Reference-based analysis of lung single-cell sequencing reveals a transitional profibrotic macrophage. *Nature Immunology* 2019;**20**:163-172.

18. Durinck S, Spellman PT, Birney E, Huber W. Mapping identifiers for the integration of genomic datasets with the R/Bioconductor package biomaRt. *Nature Protocols* 2009;**4**:1184-1191.

19. McCracken IR, Taylor RS, Kok FO, de la Cuesta F, Dobie R, Henderson BEP, Mountford JC, Caudrillier A, Henderson NC, Ponting CP, Baker AH. Transcriptional dynamics of pluripotent stem cell-derived endothelial cell differentiation revealed by single-cell RNA sequencing. *European Heart Journal* 2020;**41**:1024-1036.

20. MacAskill MG, Saif J, Condie A, Jansen MA, MacGillivray TJ, Tavares AAS, Fleisinger L, Spencer HL, Besnier M, Martin E, Biglino G, Newby DE, Hadoke PWF, Mountford JC, Emanueli C, Baker AH. Robust Revascularization in Models of Limb Ischemia Using a Clinically Translatable Human Stem Cell-Derived Endothelial Cell Product. *Mol Ther* 2018;**26**:1669-1684.

**Supplementary Table 1: Antibodies used for staining of fetal cardiac EC.**

| **Target** | **Antibody** | **Supplier** | **Catalogue Number** | **Dilution** |
| --- | --- | --- | --- | --- |
| CD31 / PECAM1 | APC anti-human CD31 | eBioscience | 17-0319-42 | 1:100 |
| CD45 / PTPRC | PE anti-human CD45 | Biolegend | 4303144 | 1:100 |

**Supplementary Table 2: Silencer Select siRNA.**

|  | **ID number** | **Catalogue Number** |
| --- | --- | --- |
| siRNA MECOM 1 | s4873 | 4392420 |
| siRNA MECOM 2 | s4872 | 4392420 |
| Negative control siRNA | NA | 4390843 |

**Supplementary Table 3: Probes used for TaqMan Assays.**

| **Target** | **ID number** |
| --- | --- |
| MECOM | Hs00602795_m1 |
| NR2F2 | Hs00819630_m1 |
| EPHB4 | Hs00174752_m1 |
| DLL4 | Hs00184092_m1 |
| HEY1 | Hs01114113_m1 |
| UBC | Hs01871556_s1 |

|  | **Target** | **Supplier** | **Catalogue Number** | **Host Species** | **Dilution** |
| --- | --- | --- | --- | --- | --- |
| **Primary** | MECOM | Abcam | ab124934 | Rabbit | 1:500 |
|  | β -actin | Santa Cruz Bio | sc-69879 | Mouse | 1:2000 |
| **Secondary** | 680RD anti-Rabbit IgG | Licor | 926-68071 | Goat | 1:10,000 |
|  | 800CW anti-Mouse IgG | Licor | 926-32210 | Goat | 1:15,000 |

**Supplementary Table 4: Antibodies used for Western Blot analysis.**

**Supplementary Figure Legends**

**Supplementary Figure 1: scRNA-seq of human fetal cardiac endothelium.**

**(A)** Feature plots showing expression of endothelial cell and non-endothelial markers. **(B)** UMAP visualisation of cells from each fetal heart sample after implementing batch correction. **(C)** Violin plots showing expression of *NPR3* (endocardial), *ACKR1* (vein/venule), *RGCC* (capillary), and *HEY1* (arterial). **(D)** GO term enrichment analysis using genes from metagene signatures C (left) and D (right). **(E)** Transcriptional signature of proliferating EC: Enrichment of signature E within proliferating EC demonstrated in SOM (left) and radar plot (middle). GO term analysis using signature E genes returns terms relating to cell proliferation (right). **(F)** Bar plot showing predicted proportion of cardiac EC in G1, G2M, and S phase of the cell cycle. **(G)** Violin plots of key marker genes defining subpopulation of fetal cardiac EC.

**Supplementary Figure 2: Profiling the transcriptional profile of subpopulations of fetal cardiac EC.**

**(A)** Heatmap showing the correlation of overall transcriptional profile of identified clusters. **(B)** Violin plots showing expression of extracellular matrix related genes (*FBLN5, ELN, FBN1*) and shear stress associated gene *KLF4*. **(C)** GO term analysis using genes differentially expressed in macrovascular EC populations (clusters 3 and 9). **(D)** Violin plots showing expression of *SMAD1*. **(E)** Heatmap showing expression of top 20 differentially expressed TFs for each cluster identified in the total dataset. TFs grouped by the cluster for which they were identified as differentially expressed in.

**Supplementary Figure 3: Trajectory inference analysis of human fetal microvascular cardiac endothelial cells.**

**(A)** Violin plots of key EC population markers shown in clusters identified in Figure 3A. **(B)** UMAP visualisation of microvascular EC with RNA velocity represented by individual arrows. **(C)** Ratio of spliced to unspliced transcripts identified for *PLVAP* and *NR2F2*. **(D)** UMAP plot of reclustered data containing cells from only endocardial, venous, and *INMT+* capillary clusters (left). Corresponding feature plots showing expression of key population markers (right). **(E)** UMAP visualisation of reanalysed data of cells from Figure 3A separated by their predicted cell cycle stage. Reanalysis conducted with the exclusion of cell cycle related genes from clustering and visualisation calculations to exclude the influence of cell cycle factors. Expression of key cluster markers shown in corresponding feature plots (right).

**Supplementary Figure 4: Dynamical changes in gene expression during human coronary endothelial cell development.**

**(A)** Spline plots showing normalised expression of *NPR3, CDH11, NR2F2, CEBPD*, *FOS,* and *MECOM* across pseudotime trajectory shown in Figure 3B. **(B)** Expression of *BMP2* in human fetal cardiac EC shown as normalised expression over pseudotime (left) and superimposed on UMAP plot from Fig3A. **(C)** Normalised expression of *DACH1* over pseudotime.

**Supplementary Figure 5: ISH validation of MECOM expression in arterial EC.**

**(A)** ISH validation of co-expression of *MECOM* with arterial TF *HEY1* in the arterial endothelium of 13- and 14-week human fetal hearts. ISH validations performed using independent fetal hearts (labelled #2, #3, and #4). **(B)** Whole heart images from ISH assay performed using negative (bacterial DapB gene) and positive (Cyclophilin B) control probes.

**Supplementary Figure 6: Expression of *MECOM* in human fetal heart scRNA-seq data.**

**(A)** UMAP visualisation of reanalysed scRNA-seq data from Suryawanshi et al^9^ obtained from 13–14-week human fetal hearts. **(B)** Feature plots showing expression of cardiomyocyte (*TNNI3*), endothelial/endocardial (*PECAM1*), leukocyte (*PTPRC*), and fibroblast markers (*PDGFRA*). **(C)** Expression of *MECOM* superimposed on UMAP plot shown in S6A.

**Supplementary Figure 7: Comparison of the endothelial cell transcriptome in the developing human and mouse heart.**

**(A)** UMAP visualisation of integrated mouse and human cardiac EC data after batch correction. **(B)** Feature plots of *INMT* and *KIT* expression in human and mouse datasets. **(C)** Heatmaps showing expression of mouse-specific markers for each subpopulation of cardiac endothelium.

**Supplementary Figure 8: siRNA mediated knockdown of MECOM in hESC-EC.**

**(A)** Expression of arterial (*SOX17, DLL4, MECOM*), venous (*NR2F2, EPHB4*), and lymphatic markers (*PROX1*) across hESC-EC differentiation. Data from McCracken et al^19^ **(B)** qRT-PCR quantification of MECOM expression following siRNA knockdown (n = 4 biological replicates). P-values were calculated using an unpaired t-test. **(C)** Western blot conformation of MECOM knockdown using siRNA MECOM 2. Blot quantified in Figure 5B. **(D)** Principal component analysis plot constructed using bulk RNA seq data obtained from hESC-EC 72 hours following transfection with either control siRNA or siRNA MECOM 1 (n = 4 biological replicates). **(E)** Expression of arterial markers (*JAG1* and *JAG2*) and pan-endothelial marker (*CDH5)*, 72 hours after MECOM knockdown (n = 4 biological replicates). Graphs in panels B and E correspond to mean ± standard error of the mean.

**Supplementary Figure 9: Uncropped western blot images from hESC-EC MECOM knockdown.**

Uncropped western blot images demonstrating abundance of MECOM following knockdown using **(A)** siRNA MECOM 1. **(B)** siRNA MECOM 2.


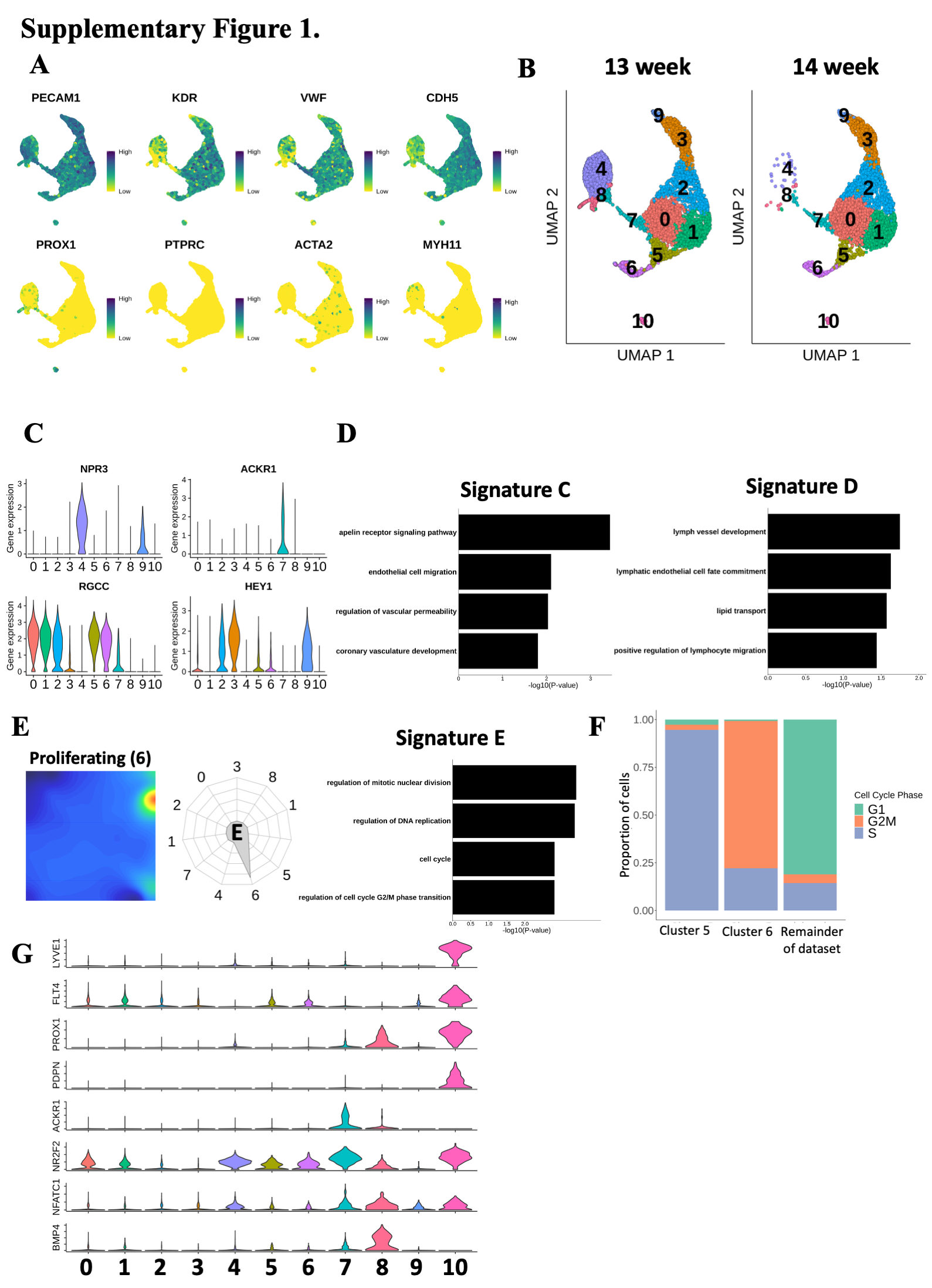

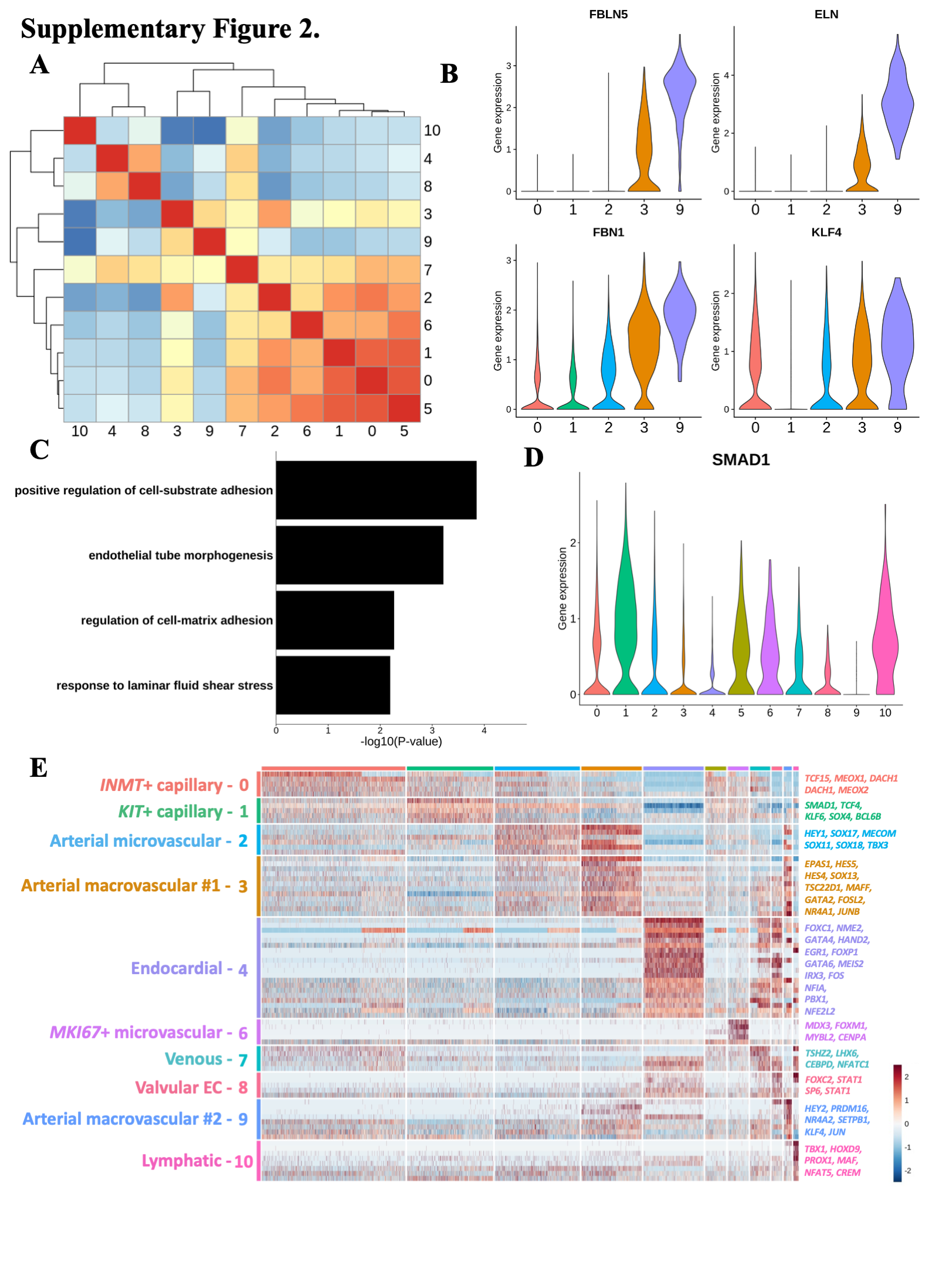


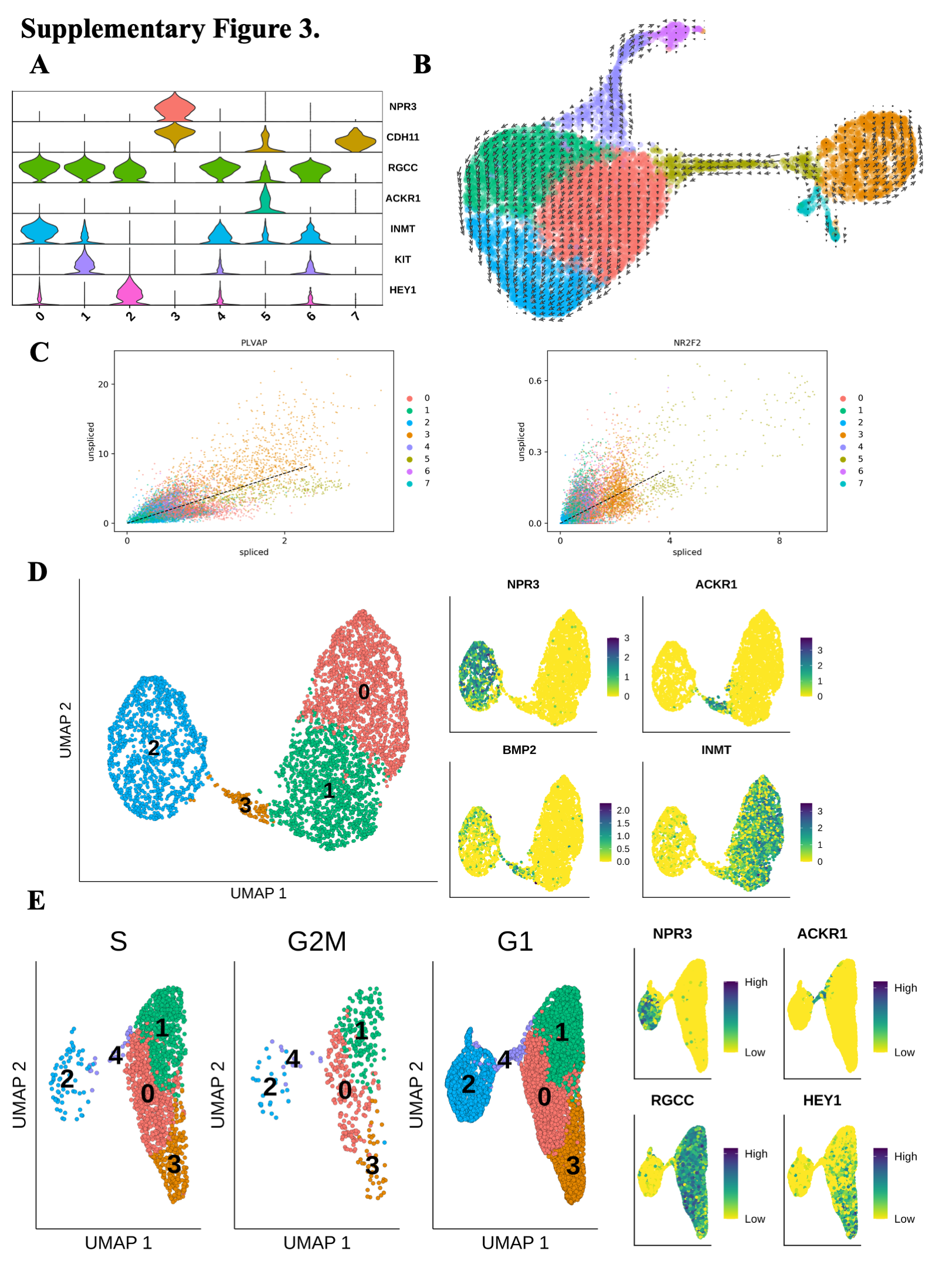


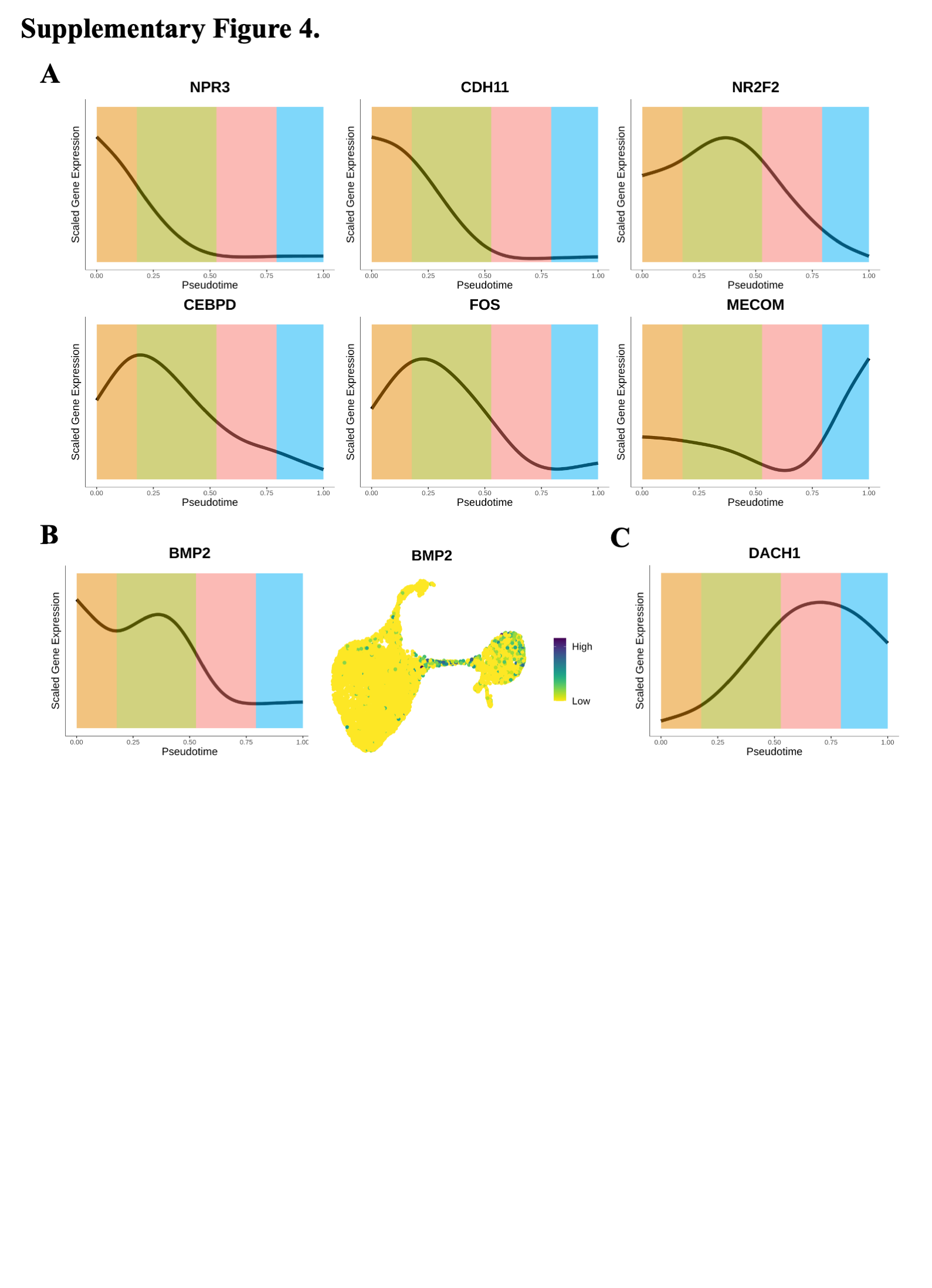


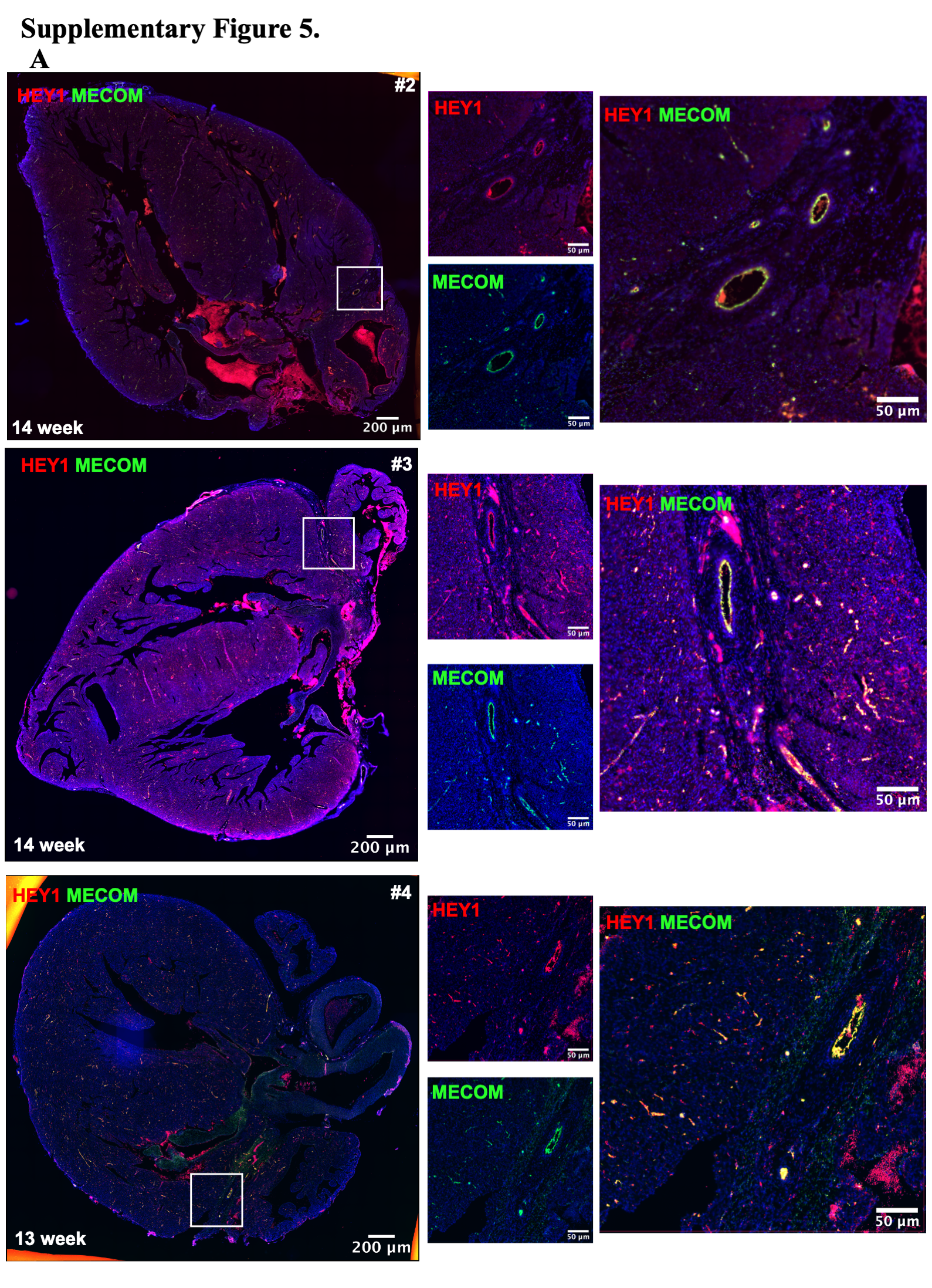


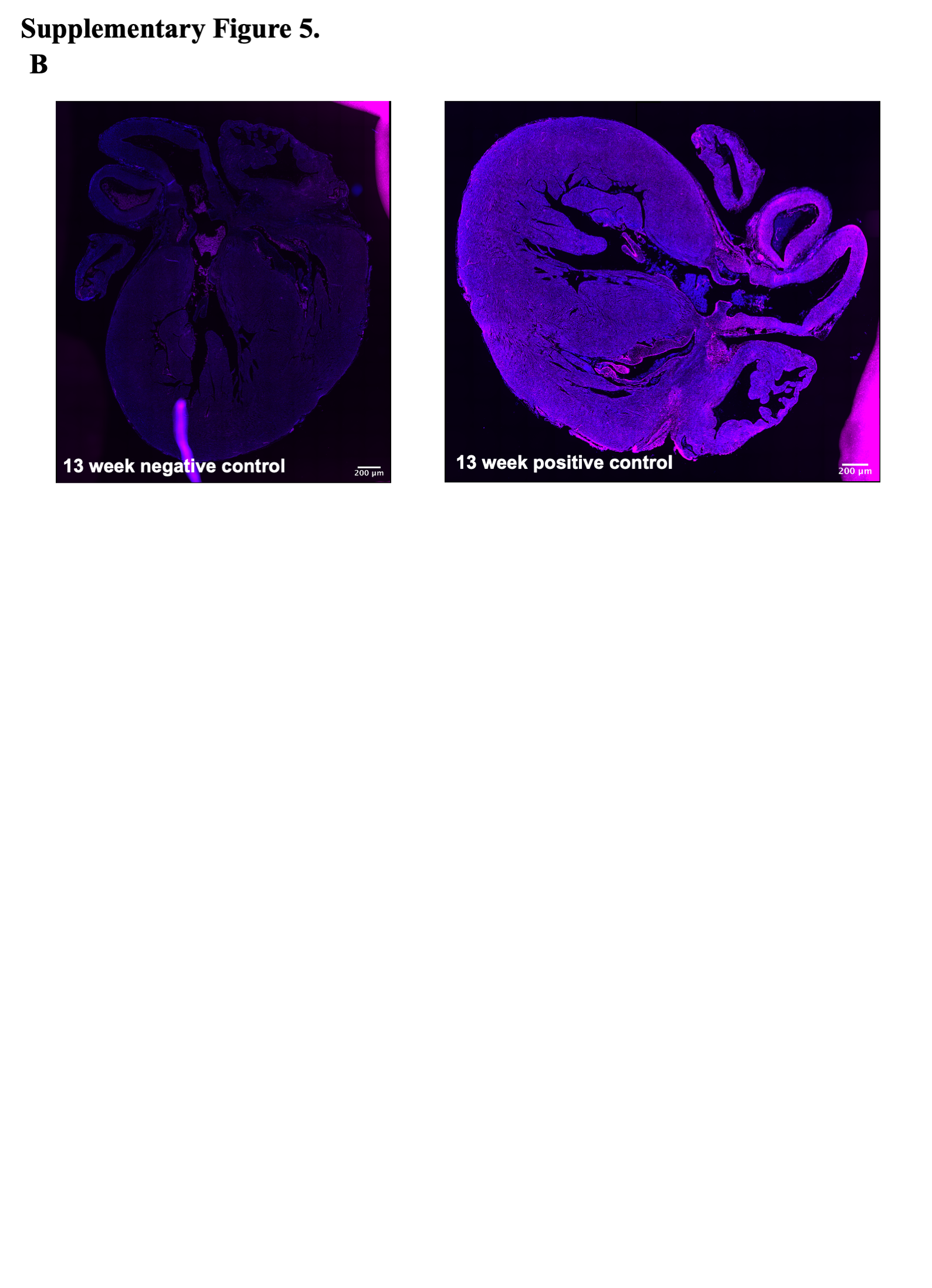


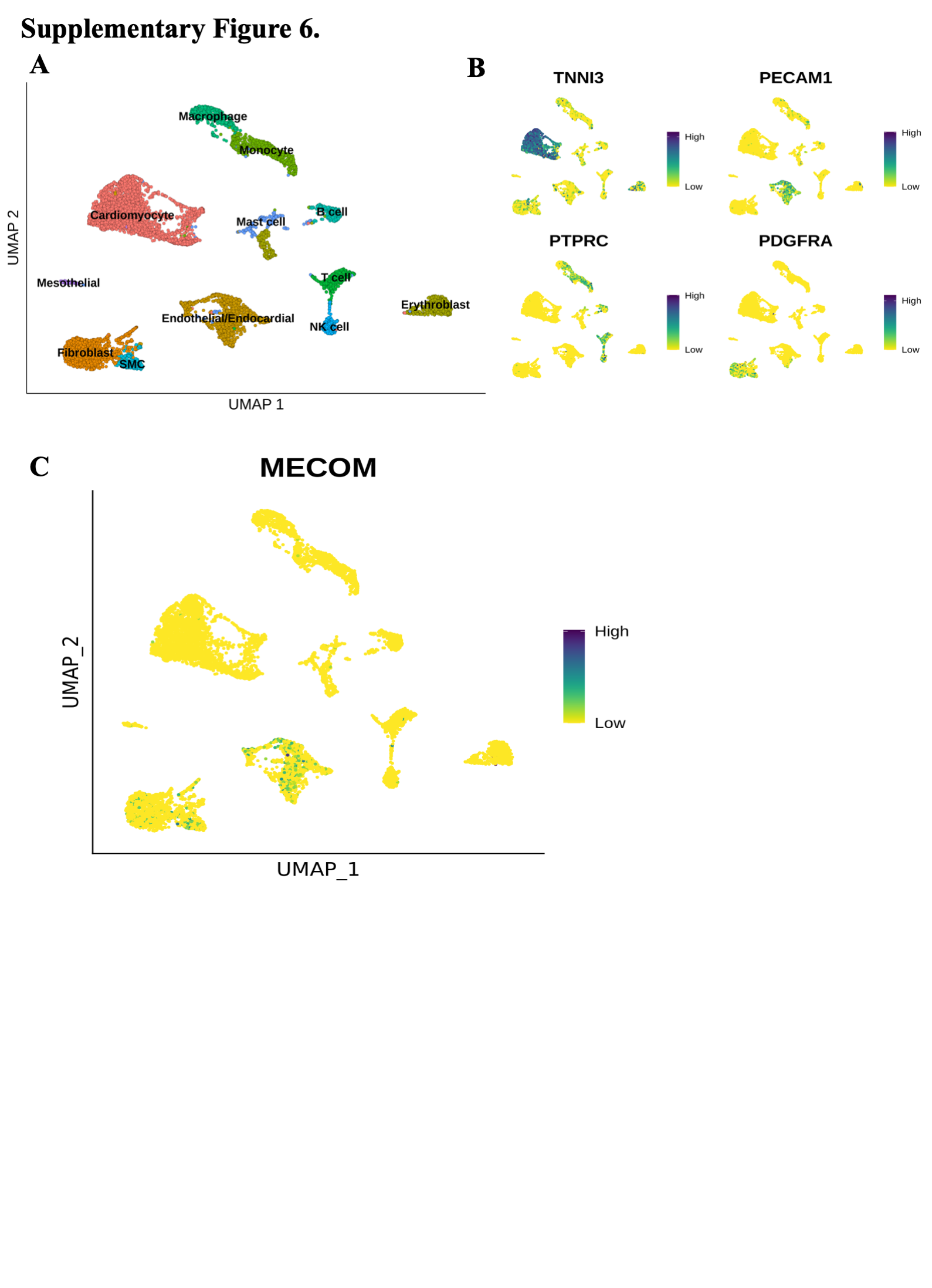


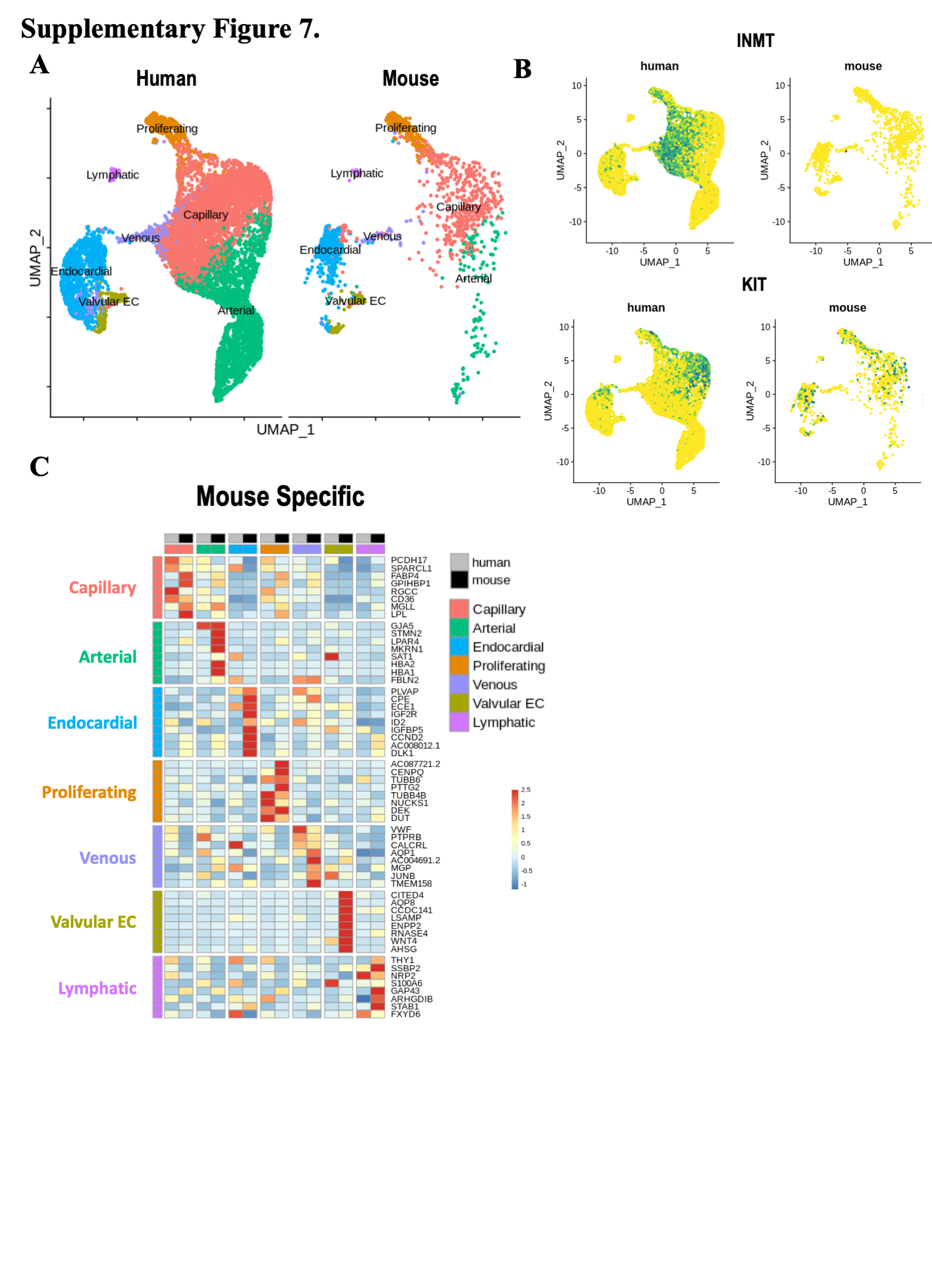


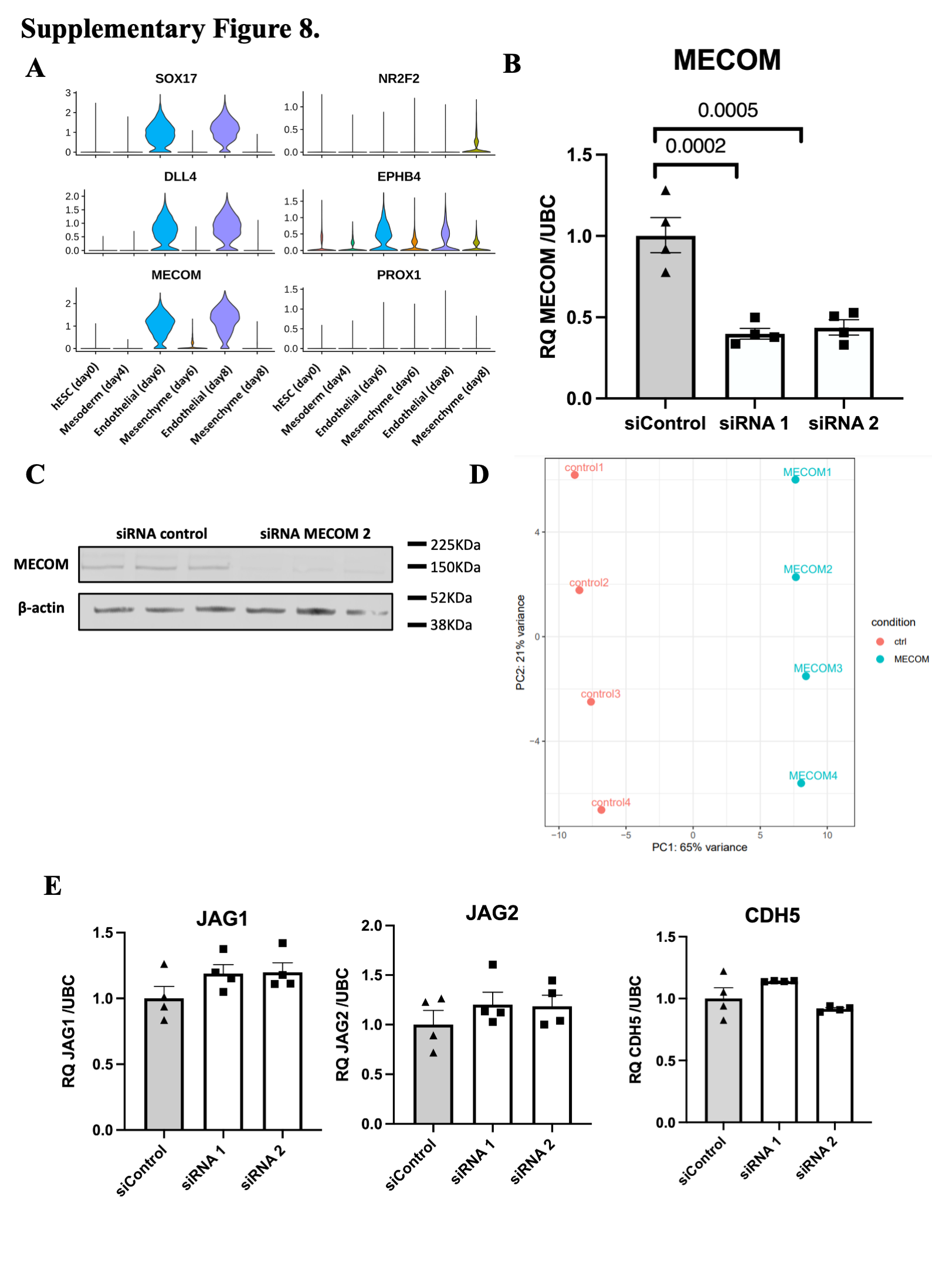


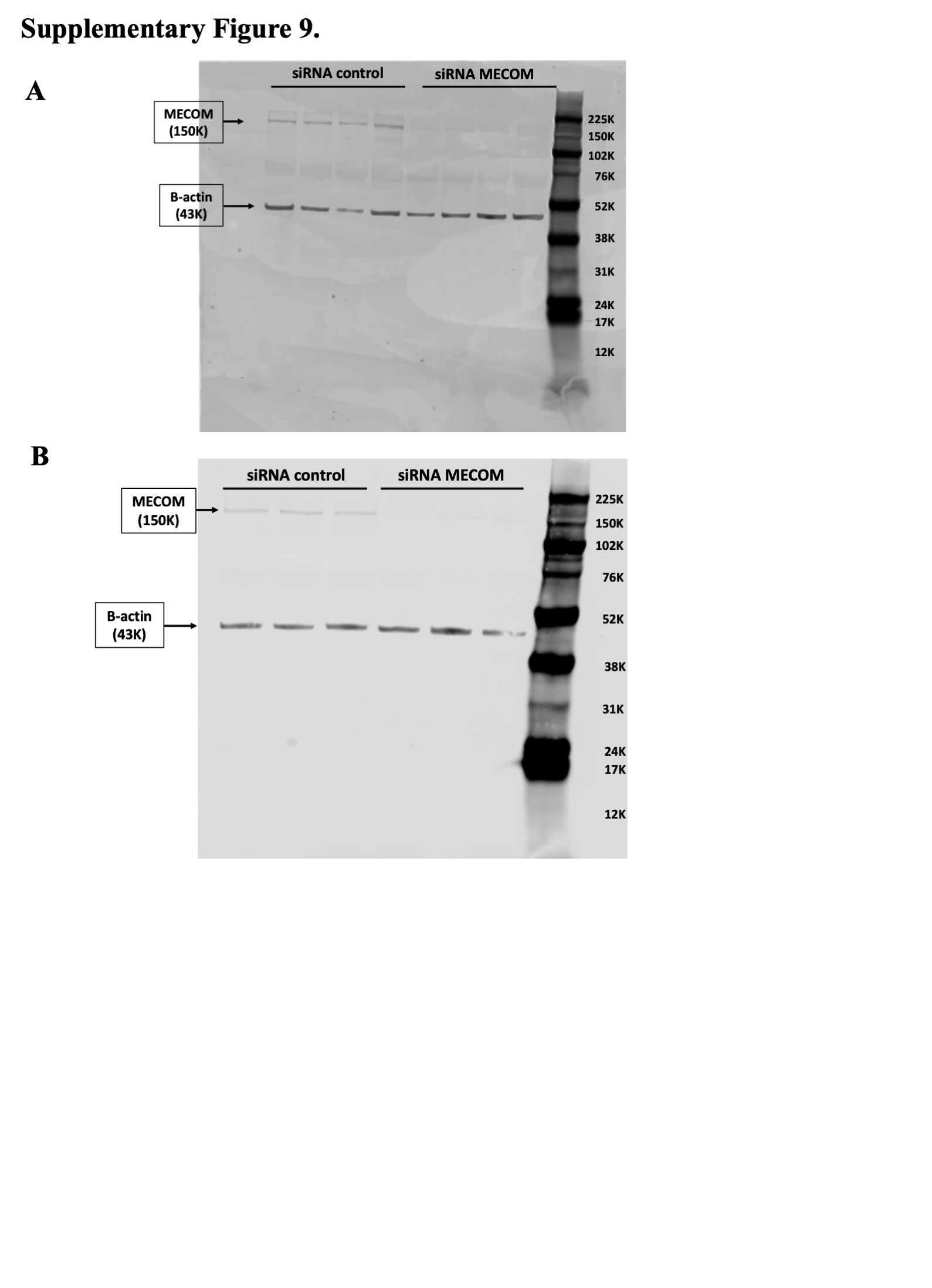

Supplement: cvac023_Supplementary_Data [file cvac023_supplementary_data.zip › McCracken et al (2022) CVR supplementary 090222 v2.docx]
