# Supplementary figures and images for: Mapping the developing human cardiac endothelium at single-cell resolution identifies MECOM as a regulator of arteriovenous gene expression
Source: Cardiovasc Res. 2022 Feb 25;118(14):2960–72. doi: 10.1093/cvr/cvac023 (PMC9648824; doi:10.1093/cvr/cvac023)

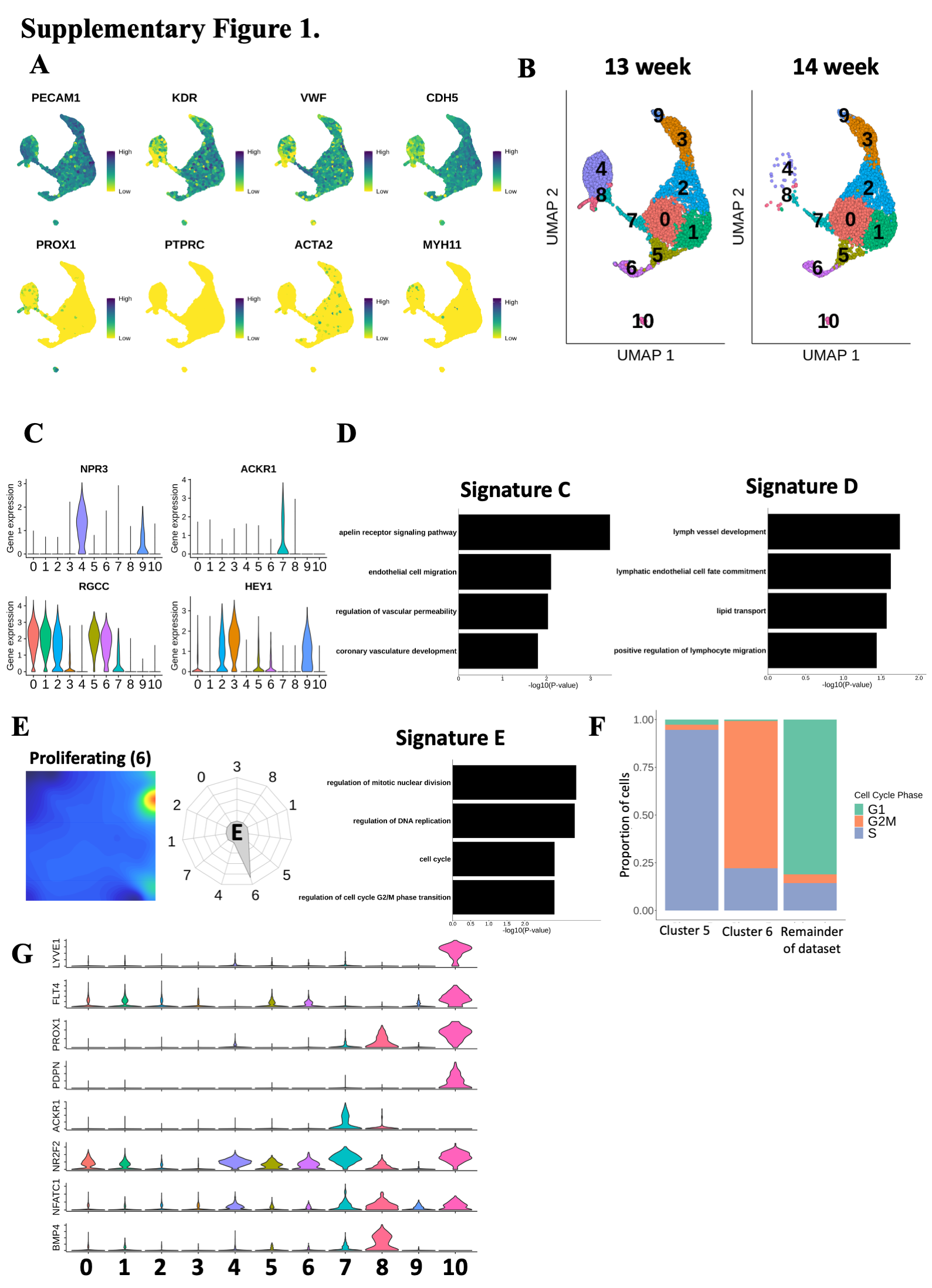

Supplement: cvac023_Supplementary_Data [file cvac023_supplementary_data.zip › Supplementary_Figure_1.tiff]

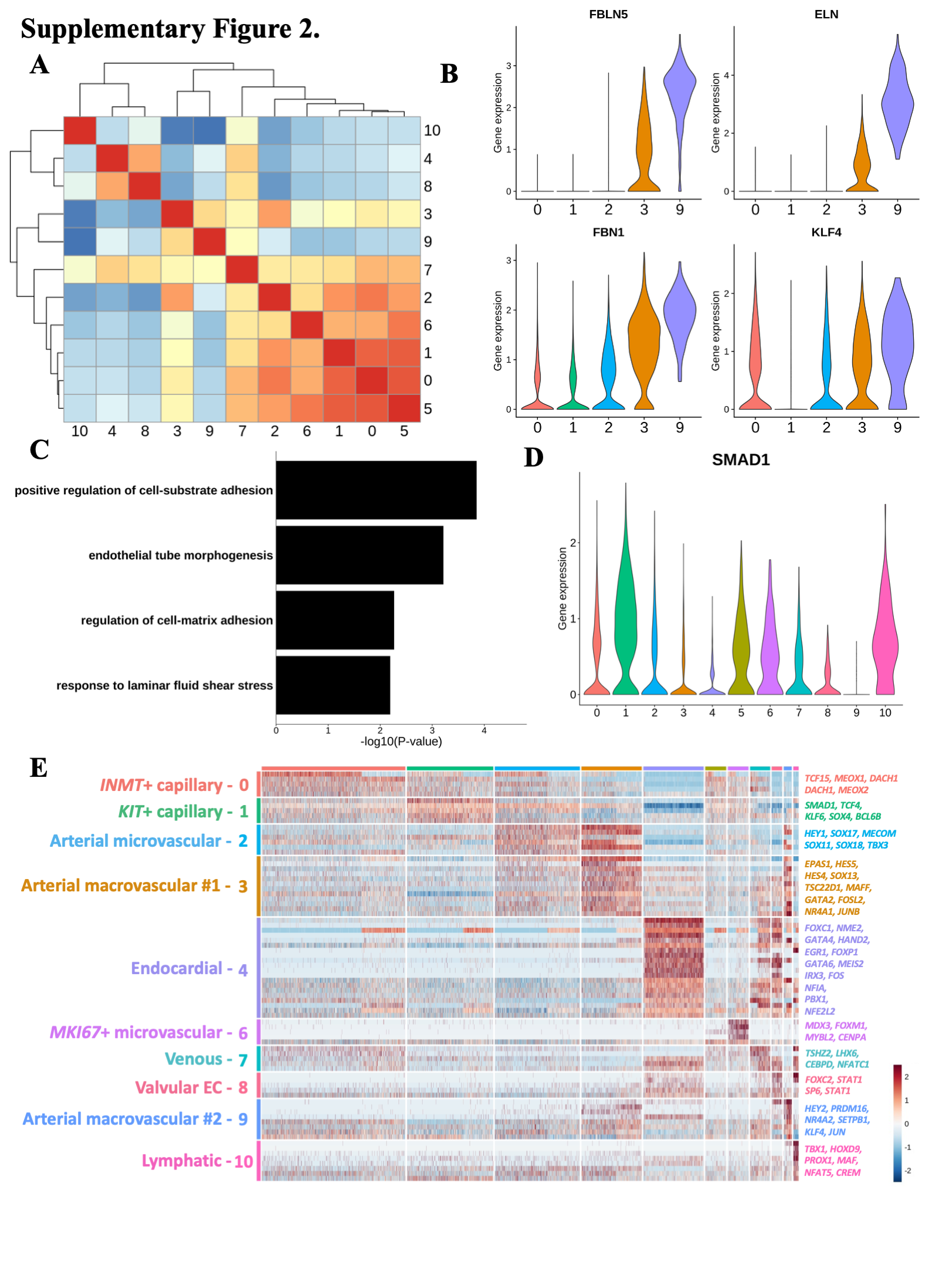

Supplement: cvac023_Supplementary_Data [file cvac023_supplementary_data.zip › Supplementary_Figure_2.tiff]

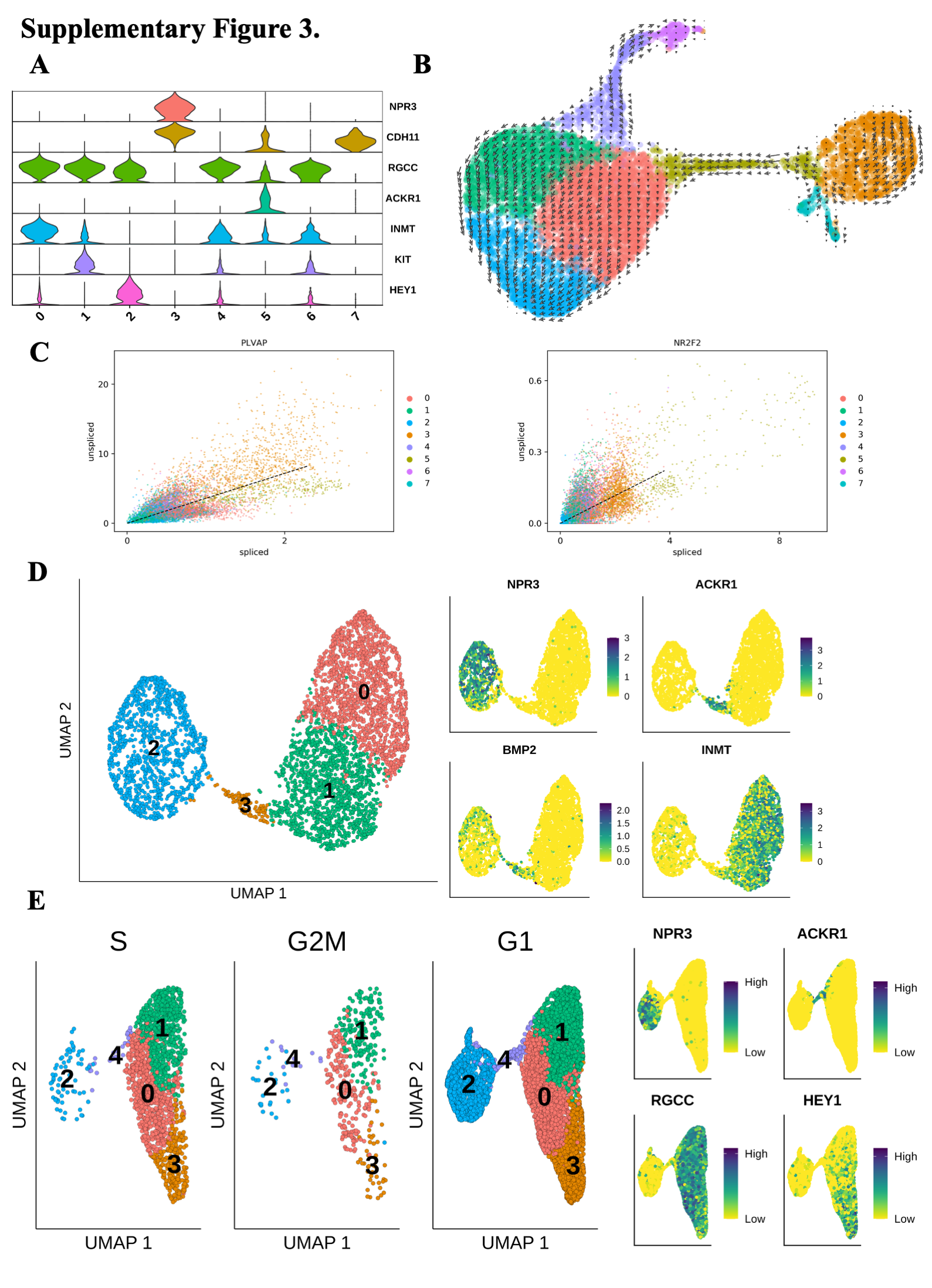

Supplement: cvac023_Supplementary_Data [file cvac023_supplementary_data.zip › Supplementary_Figure_3.tiff]

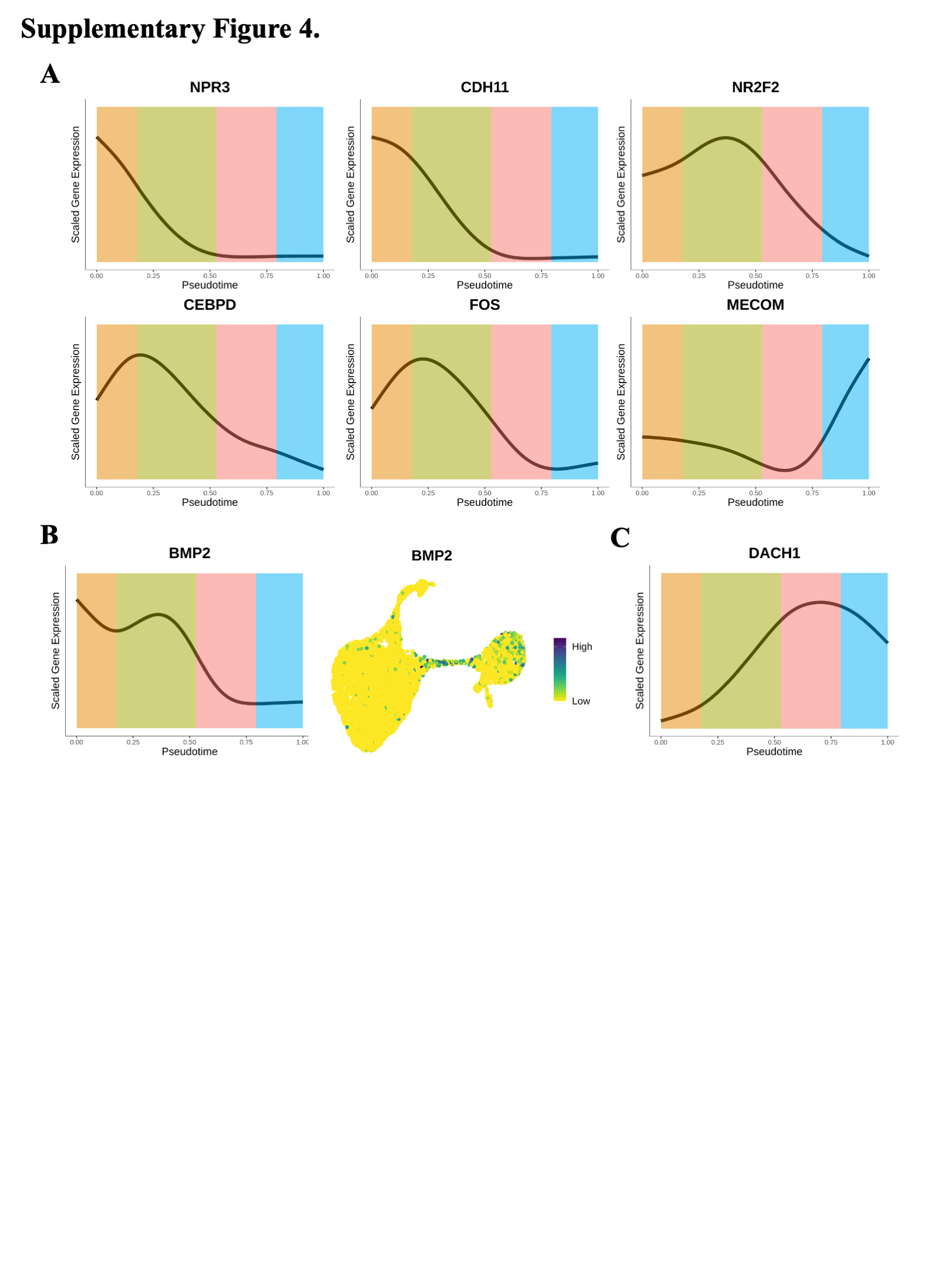

Supplement: cvac023_Supplementary_Data [file cvac023_supplementary_data.zip › Supplementary_Figure_4.tiff]

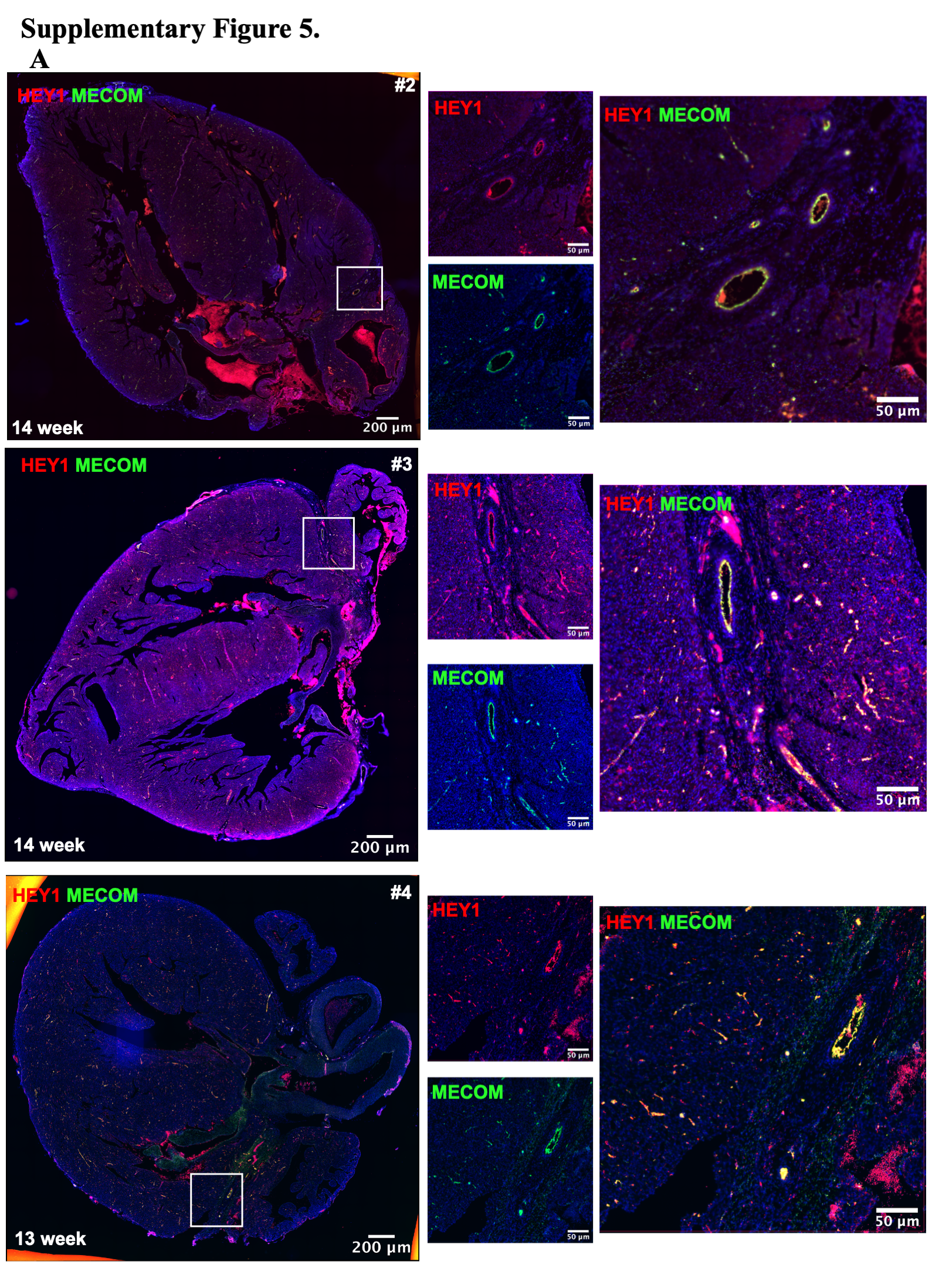

Supplement: cvac023_Supplementary_Data [file cvac023_supplementary_data.zip › Supplementary_Figure_5A.tiff]

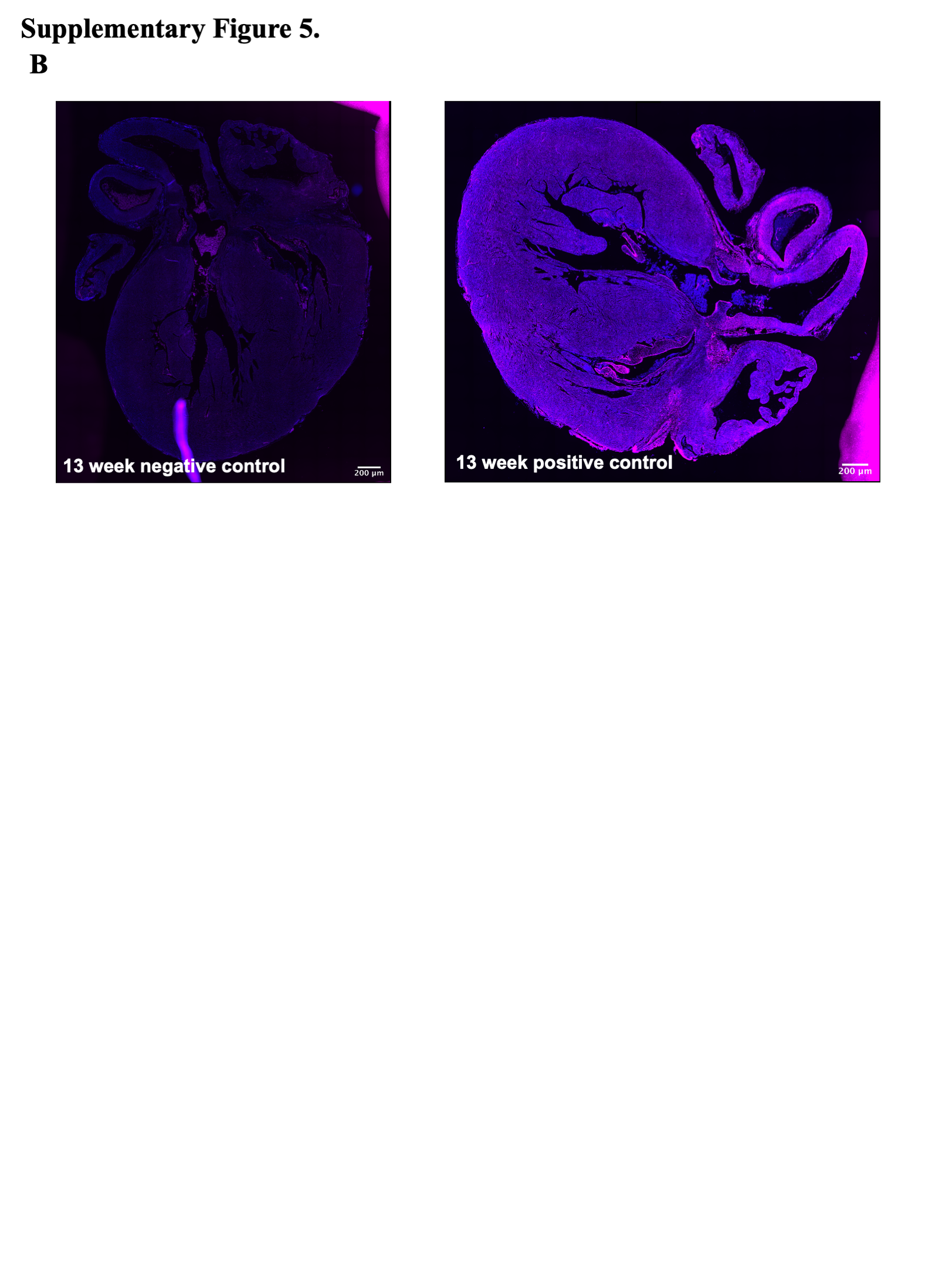

Supplement: cvac023_Supplementary_Data [file cvac023_supplementary_data.zip › Supplementary_Figure_5B.tiff]

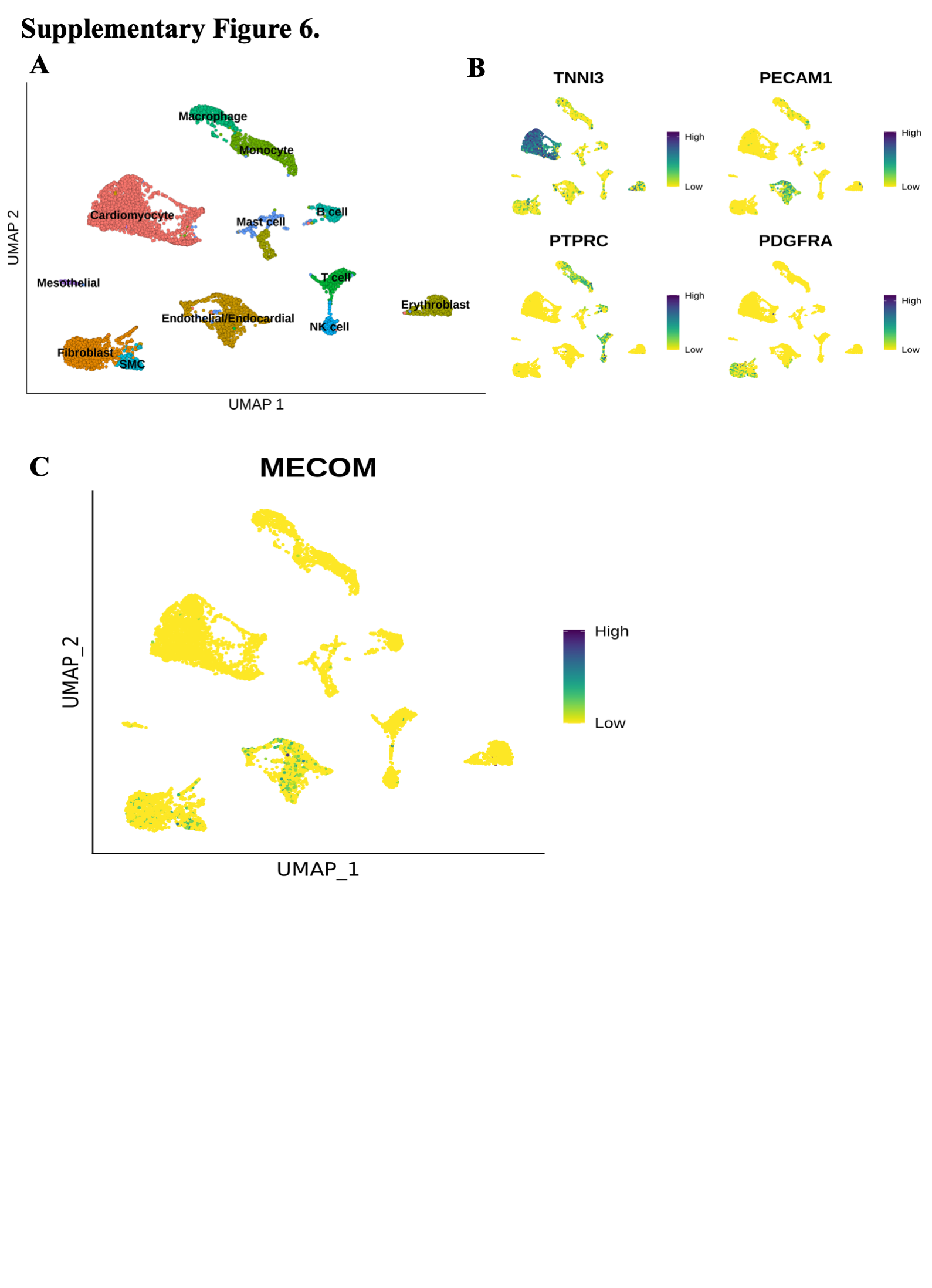

Supplement: cvac023_Supplementary_Data [file cvac023_supplementary_data.zip › Supplementary_Figure_6.tiff]

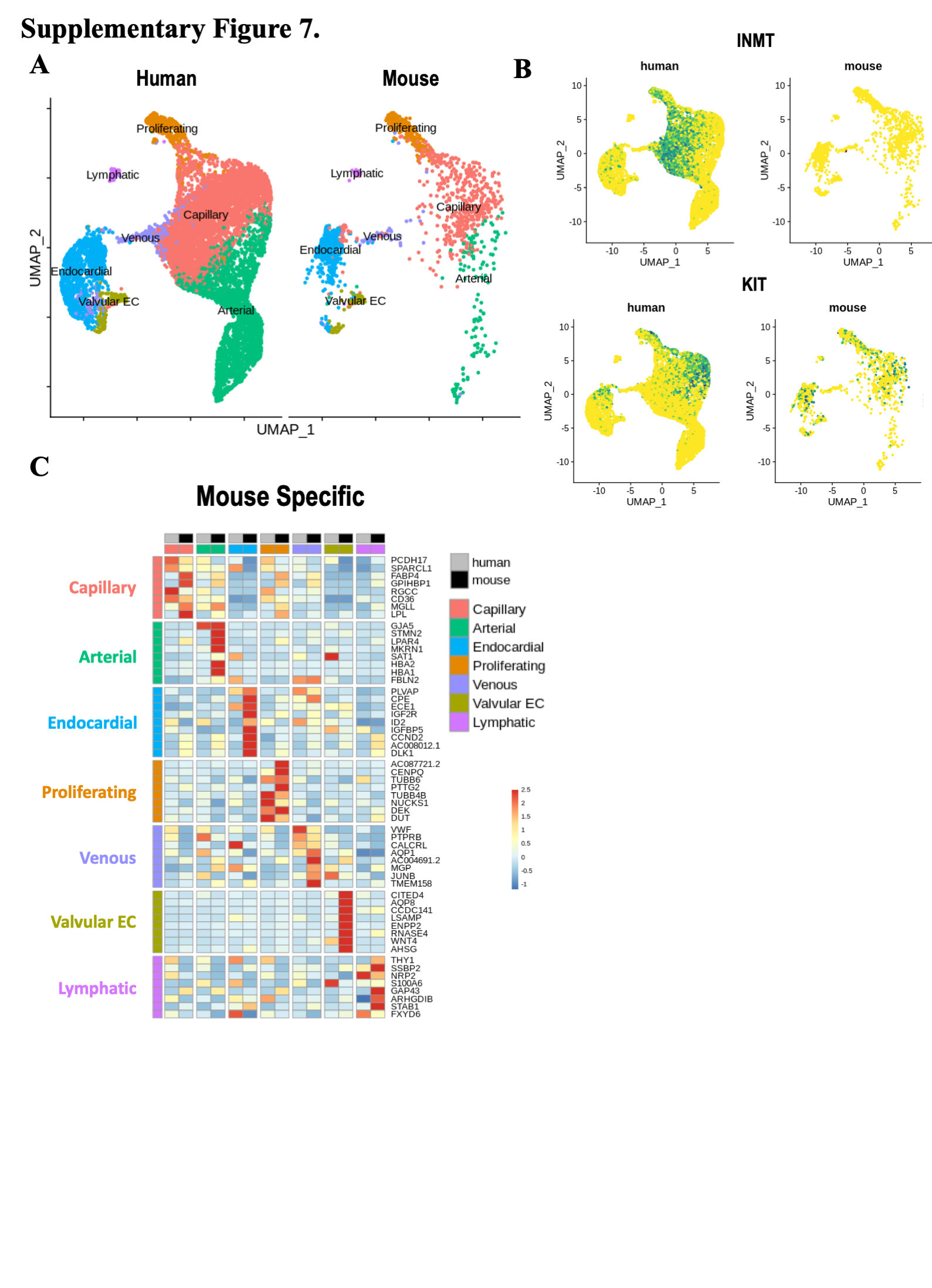

Supplement: cvac023_Supplementary_Data [file cvac023_supplementary_data.zip › Supplementary_Figure_7.tiff]

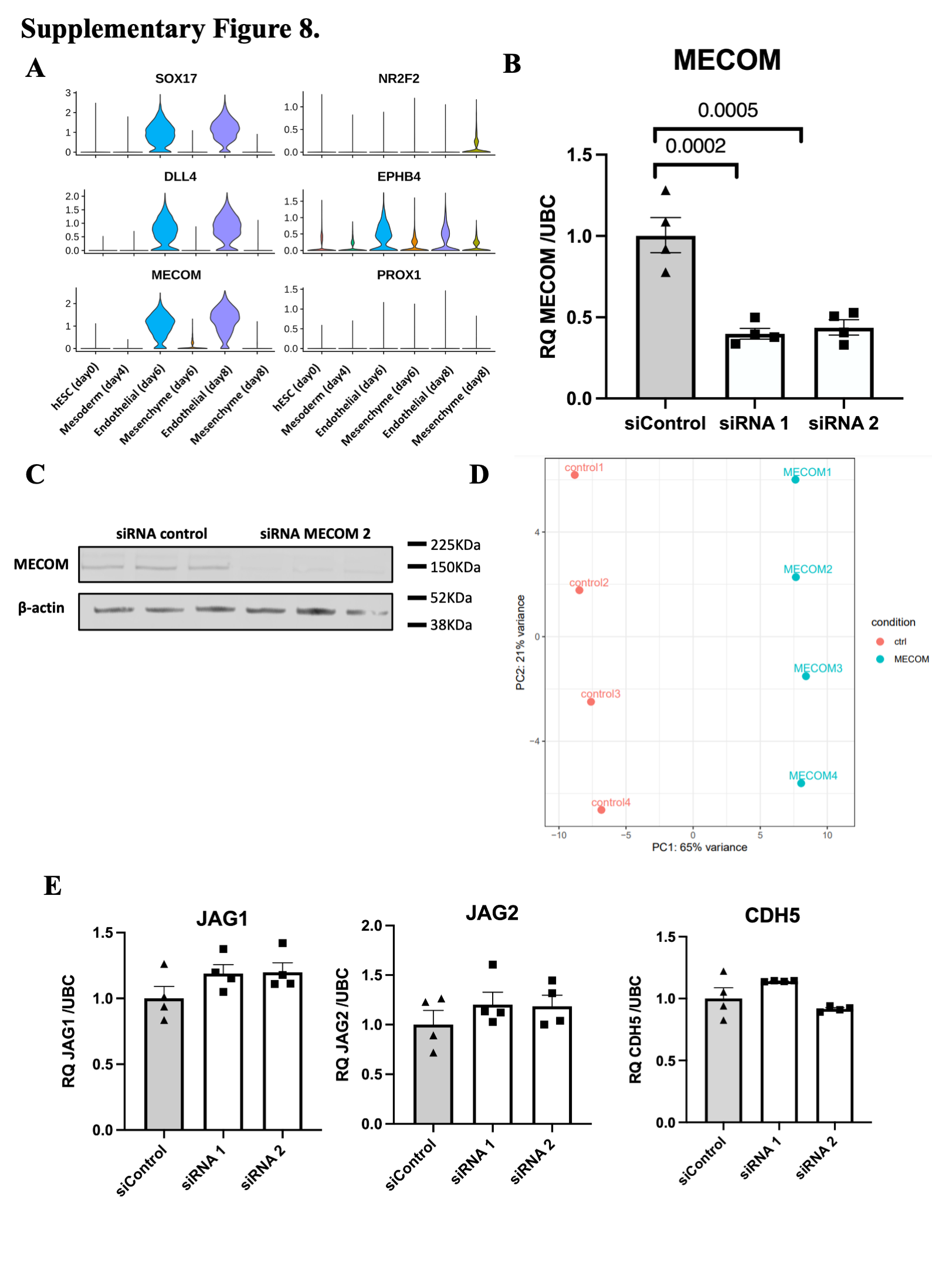

Supplement: cvac023_Supplementary_Data [file cvac023_supplementary_data.zip › Supplementary_Figure_8.tiff]

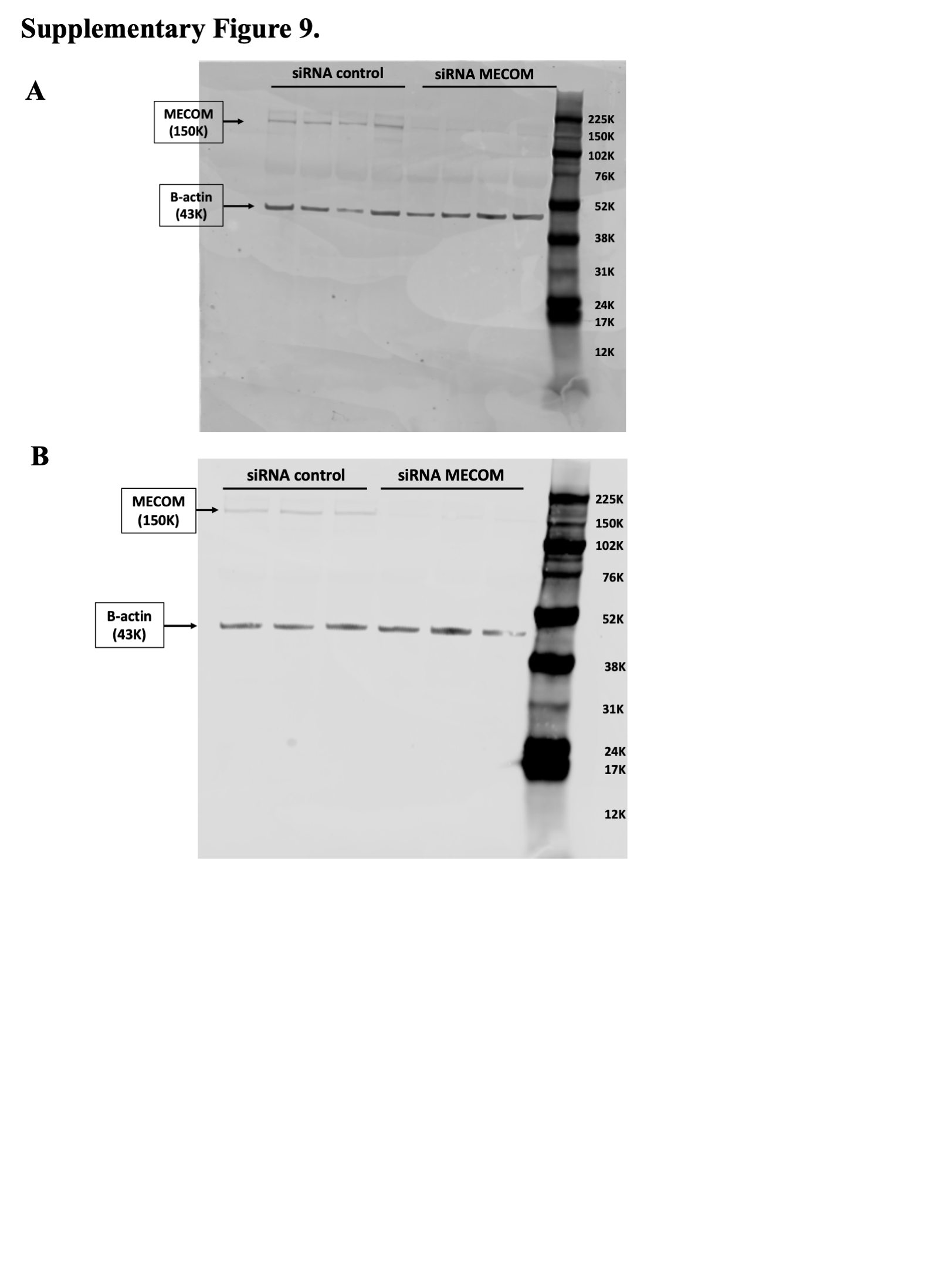

Supplement: cvac023_Supplementary_Data [file cvac023_supplementary_data.zip › Supplementary_Figure_9.tiff]
